# Testosterone upregulates glial cell line-derived neurotrophic factor (GDNF) and promotes neuroinflammation to enhance glioma cell survival and proliferation

**DOI:** 10.1186/s41232-023-00300-7

**Published:** 2023-10-13

**Authors:** Kouminin Kanwore, Konimpo Kanwore, Xiaoxiao Guo, Ying Xia, Han Zhou, Lin Zhang, Gabriel Komla Adzika, Adu-Amankwaah Joseph, Ayanlaja Abdulrahman Abiola, Peipei Mu, Piniel Alphayo Kambey, Marie Louis N’dzie Noah, DianShuai Gao

**Affiliations:** 1 Public Experimental Research Center, Department of Neurobiology and Anatomy, Xuzhou Medical University, Xuzhou, Jiangsu 221004 China; 2 Mixed Faculty of Medicine and Pharmacy, University of Lomé, Lomé, Togo; 3 Department of Physiology, Xuzhou Medical University, Xuzhou, Jiangsu China

**Keywords:** Glioma, Testosterone, GDNF, Cyclophilin A, Neuroprotection, Neuroinflammation

## Abstract

**Background:**

Testosterone contributes to male organism development, such as bone density, muscle development, and fat repartition. Estrogen (derived from testosterone) also contributes to female reproductive system development. Here, we investigated the effect of testosterone on glioma cells and brain neuron inflammation essential for cancer development and progression.

**Methods:**

The human astrocyte and glioma cell lines were treated with 6 ng/ml exogenous testosterone in vitro. We performed cell counting kit-8, transwell, and wound healing assays to determine the effect of testosterone on glioma cell proliferation, migration, and invasion. The glioma cells were injected into the xenograft and treated with 5 µl concentrated testosterone. Transcriptional suppression of glial cell line-derived neurotrophic factor (GDNF) was performed to evaluate brain neuron inflammation and survival. The tumor tissues were assessed by hematoxylin–eosin staining and immunohistochemistry.

**Results:**

Testosterone upregulates GDNF to stimulate proliferation, migration, and invasion of glioma cells. Pathologically, the augmentation of GDNF and cyclophilin A contributed to neuroprotection when treated with testosterone. Our investigation showed that testosterone contributes to brain neuron and astrocyte inflammation through the upregulation of nuclear factor erythroid 2-related factor 2 (NRF2), glial fibrillary acid protein (GFAP), and sirtuin 5 (SIRT5), resulting in pro-inflammatory macrophages recruitments into the neural microenvironment. Mechanically, testosterone treatment regulates GDNF translocation from the glioma cells and astrocyte nuclei to the cytoplasm.

**Conclusion:**

Testosterone upregulates GDNF in glioma cells and astrocytes essential for microglial proliferation, migration, and invasion. Testosterone contributes to brain tumor growth via GDNF and inflammation.

**Graphical Abstract:**

The contribution of testosterone, macrophages, and astrocytes, in old neuron rescue, survival, and proliferation. During brain neuron inflammation, the organism activates and stimulates the neuron rescue through the enrichment of the old neuron microenvironment with growth factors such as GDNF, BDNF, SOX1/2, and MAPK secreted by the surrounding neurons and glial cells to maintain the damaged neuron by inflammation alive even if the axon is dead. The immune response also contributes to brain cell survival through the secretion of proinflammatory cytokines, resulting in inflammation maintenance. The rescued old neuron interaction with infiltrated macrophages contributes to angiogenesis to supplement the old neuron with more nutrients leading to metabolism activation and surrounding cell uncontrollable cell growth.

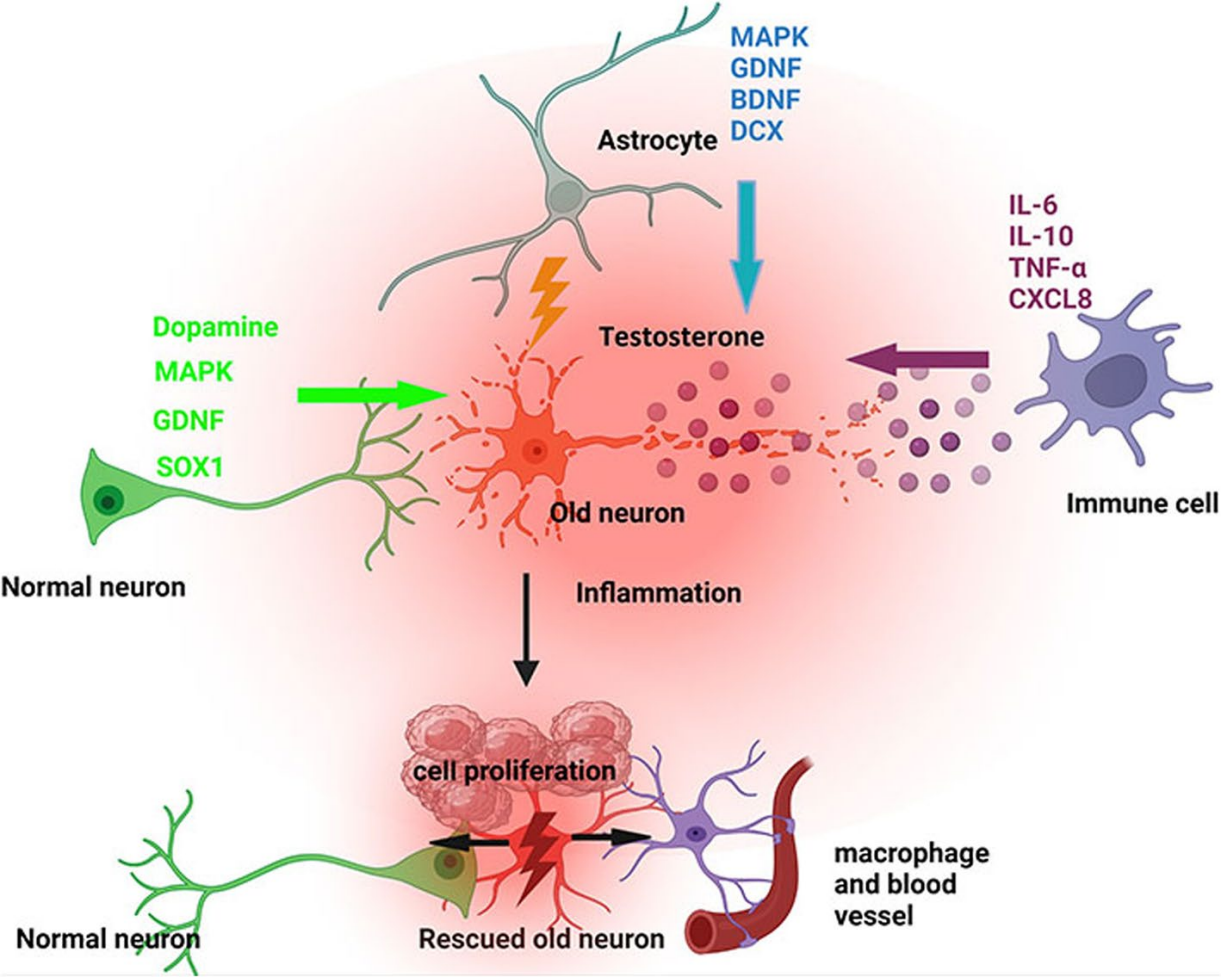

**Supplementary Information:**

The online version contains supplementary material available at 10.1186/s41232-023-00300-7.

## Background

According to the World Health Organization (WHO), males are predisposed to glioblastoma with the lowest survival rate compared to females [1, 2]. The hormonal differences between males and females start during embryogenesis, influenced by chromosome X for females and Y for males that stimulates the expression of the genes involved in sex differentiation, such as sex-determining region Y (SRY) family proteins, including SRY-box (SOX) genes [3, 4]. The upregulation of the SOX gene regulates male sex differentiation more than females [4]. An increase in SOX-1 expression was reported in brain cancer, contributing to brain tumor development [5]. Consistently, SOX-1 is also reported to contribute to cervical tumor suppression [6]. Munkley J et al. 2015 and McHenry J et al. 2014 demonstrated an interaction between SOX-1 and testosterone at mRNA level [7, 8]. Kanwore K. et al. 2020 reported a possible interaction between glial cell line-derived neurotrophic factor (GDNF) and SOX-1 that may regulate glioblastoma malignancy [9]. GDNF was first isolated in 1993 by Lin et al. and shown to contribute to brain cancer development [10]. Several studies have described the role of GDNF in central nervous system diseases such as brain cancer and Parkinson's disease [11–14]. Rodrigez-Lozano, DC et al., in 2019, reported that testosterone promotes glioblastoma cell proliferation, invasion, and migration [15]. In fact, the binding of testosterone to the androgen receptor located on cancer cells triggers the induction of an internal signal that activates the transcription of DNA into RNA through the androgen response element, thus increasing the expression of oncogenes such as GDNF and SOX-1 necessary for cell proliferation [16, 17]. The activation and overexpression of androgen receptors improve glucose uptake and metabolism, contribute to cancer cell proliferation, and increase tumor volume [18]. Typically, in glioma patients, there is an increase in muscular and fat mass loss and the transformation of cholesterol into steroids, which is transformed into testosterone and used by cancer cells to proliferate [19].

In this study, we hypothesized that testosterone regulates neuroinflammation and GDNF upregulation to favor tumor formation and development. We investigated the role of testosterone on oncogenes differential expression and their effects on glioma cell proliferation, migration, and invasion. Our study demonstrated that testosterone activates GDNF to protect the glioma cell line by inducing neuro-inflammation necessary for tumor development. The aim of this study is not the evaluation of testosterone’s role in sex differences in glioma biology. We presented the data of U251 and human astrocytes only because the U87 glioma cell lines have been reported to have altered properties and are unstable. LN229 was able to grow a tumor in immunocompromised or nude mice only. The LN229 cell lines could not grow a tumor in normal balb C mice, explaining our choice to focus on human astrocyte and U251 glioma cell lines. However, we provided the results of the western blot using these glioma cell lines (U87, U251, LN229, and HA) in the Supplementary section.

## Materials and methods

### Antibodies

**Table:** 

Antibody	Cat. Number	Company
Beta-actin	#4967	Cell signaling technology
Beta-tubulin	#2146	Cell signaling technology
NRF2	#8882	Cell signaling technology
GDNF	ab18956	Abcam
GFAP	#3670	Cell signaling technology
IBA 1	10,904–1-AP	Proteintech
ERK1/2	11,257–1-AP	Proteintech
pERK1/2	28,733–1-AP	Proteintech
Cyclophilin A	10,720–1-AP	Proteintech
CD86	Ab119857	Abcam
CD68	Ab213363	Abcam
iNOS	#2982	Cell signaling technology
Survivin	#2803	Cell signaling technology
CD40L	16,669–1-AP	Proteintech
Secondary antibodies	Alexa fluor 488, AB_2889374, Alexa fluor 594, SA00006-8	Proteintech

### Cell culture

The human astrocyte (HA), LN229 (RRID: CVCL_0393), U87 (RRID: CVCL_0022), and U251 (RRID: CVCL_0021) cell lines authenticated by American Type Culture Collection were obtained. Before culturing the U251 and HA in the DMEM medium, the cells were starved using no glucose, no glutamine, no sodium pyruvate, and no phenol medium supplemented with 1% penicillin and 10% fetal bovine serum for 24 h. This was done to stimulate nutrient intake. The U251 and HA were cultured in the DMEM medium and treated with testosterone (57–85-2, Sigma-Aldrich) for 48 h. The experimental groups were incubated in DMEM high glucose medium supplemented with 1% penicillin, 10% fetal bovine serum at 37 °C, and 6 ng/ml testosterone (57–85-2, Sigma-Aldrich). The control (untreated) were incubated in DMEM high glucose medium supplemented with 1% penicillin and 10% fetal bovine serum at 37 °C without testosterone. All the experiments were performed with mycoplasma-free cells.

### Next-generation sequence

We proceeded to the pattern of gene expression across human U251 and HA using the next-generation sequence. Data were processed and transformed using the EdgeR:log2(CPM + c) function of the online tool iDEP.96 (iDEP.96 (sdstate.edu)). Differential expression analysis was performed using the DESeq2 function of iDEP.96, where the expression profiles of GDNF overexpressed cells and controls were compared to identify the differential expression genes (DEGs). Adjusted* p* values were calculated using *t* tests. Genes from each sample with the following criteria were retained: (1) a log_2_ FC > 1 and (2) an adjusted *p* < 0.05.

### Coculture U251, HA, and RAW 264.7

The human astrocyte (HA), U251 (RRID: CVCL_0021), LN229 (RRID: CVCL_0393), and mouse RAW 264.7 (RRID: CVCL-0493), all obtained from American Type Culture Collection, were respectively cultured in two loculi separated with a membrane of (0.6 µM) to allow fluid traffic between the two chambers. In another dish, we mixed U251 and Raw 264.7 cells. We repeated the same procedure for HA coculture with Raw 264.7. After 48 h, imaging was done with an Olympus microscope.

### Reverse transcription-polymerase chain reaction (PCR)

The RNA was isolated using Trizol (Life technology) following the steps indicated by the manufacturer. The RNA was transformed into cDNA via reverse transcription using a cDNA synthesis kit (MedChemExpress HY-K0510A, MCE), and the cDNA was polymerized using SYBR Green MasterMix (MedChemExpress HY-K0510A, MCE) mixed with primers. The results were read using Roche Lightcycler 480-II.

**Table:** 

Gene	Primers
GDNF	F: CAGTGACTCCAATATGCCTGA R: CCGCTTGTTTATCTGGTGAC
SOX-1	F: AAAACCCCAAGATGCACAACTC R: TCTTGAGCAGCGTCTTGGTCT
Prostaglandin (PTGIR)	F: GCACGAGAGGATGAAGTTTAC R: AGGATGGGGTTGAAGGCGTT
Androgen receptor (AR)	F: CCTGGCTTCCGCAACTTACAC R: GGACTTGTGCATGCGGTACTCA
Interleukin-6 (IL-6)	F: CTTGGGACTGATGCTGGTGACA R: GCCTCCGACTTGTGAAGTGGTA
COX-2	F: CCTGTGTTCCACCAGGAGAT R: CCCTGGCTAGTGCTTCAGAC
GFAP	F: ACATCGAGATCGCCACCTAC R: CCTTCTGACACGGATTTGGT

### Western blot

Total protein lysates were obtained using total protein extraction reagents. Protein samples were analyzed by western blotting using standard procedures. Briefly, protein extracts were separated by SDS-PAGE, transferred to PVDF membranes, and blocked with 5% skim milk in TBST for 2 h. The membranes were incubated with the following primary antibodies (GDNF, NRF2, Cyclophilin A, ERK1/2) overnight at 4 °C. Next, the membranes were probed with secondary antibodies at room temperature for 2–3 h. The bands were then scanned with an imaging system (LICOR CXL) and quantified by ImageJ software version 1.45S.

### Immunofluorescence: cell and tissue

The GCL grown on a confocal dish and xenograft organ tissues were fixed with 4% paraformaldehyde for 30 min and permeabilized with 1% Triton X-100 for 10 min. After blocking for 30 min using 5% BSA, cells were incubated in primary antibody (GDNF) diluted in PBS overnight at 4 °C. Then, cells were incubated with secondary antibodies for 2 h at 37 °C. Nuclei were stained with Hoechst and/or DAPI. For the tissue immunostaining, we proceeded to deparaffinize the tissue in xylene after antigenic retrieval using heated citrate buffer at 121 °C for 30 min, fixed with formalin and blocked in 5% bovine serum albumin. The tissues were incubated with the primary antibody (GFAP, CD86, CD68, GDNF) overnight at 4 °C. After rinsing with PBS, the secondary antibody was added at room temperature for 3–4 h and DAPI for 1 h. Fluorescence images were captured under a confocal microscope at the same exposure intensity.

### Invasion assay

The 24-well plate was coated with agarose solution (1%) on which the glioma cell lines (4000 cells/well) were incubated and covered with DMEM high glucose medium for 24 h. After 24 h, the glioma cell line formed spheroids which were transferred in another invasion matrix composed of Matrigel and DMEM medium for 72 h. The images were taken using an Olympus microscope.

### Transwell

Cell invasion was determined by Transwell matrigel assay. Cells were seeded on the upper Matrigel-coated transwell chamber in 24-well plates. Cells were cultured with a serum-free medium in the upper chamber, and DMEM containing 10% FBS was added to the lower chamber. After incubation for 48 h, the non-invaded cells were gently wiped with a cotton swab, and invaded cells were fixed with 4% paraformaldehyde and stained with 0.1% crystal violet. The stained cells were visualized by a light microscope and counted by ImageJ.

### Wound-healing assay

To assess the cell migration, wound healing assays were performed as previously described [20]. Briefly, the confluent cell monolayer was scratched with a 100-μl pipette tip to create a wound. After washing with PBS, cells were cultured in normoxia conditions for 24 h and 48 h and observed under a light microscope. The wound area was calculated using ImageJ to assess the rate of cell migration.

### Cell counting kit 8 (CCK-8)

Cell viability was analyzed by Cell Counting Kit‐8 (CCK8, Beyotime, Shanghai, China) according to the manufacturer’s protocols. Cells were seeded and cultured at a density of 5 × 10^3^/well in 100 μL of the medium into 96‐well microplates (Corning, USA). Then, the cells were treated as indicated in the cell culture section. After treatment for 48 h, 10 μL of CCK‐8 reagent was added to each well and then cultured for 2 h. All experiments were performed in triplicate. Using a microplate reader (Bio-Rad, Hercules, CA, USA) and wells devoid of cells as “blanks,” the absorbance was measured at 450 nm.

### 5-Ethynyl-2’-Deoxyuridine (EdU) assay

EdU assay was conducted to examine the cell proliferation using the EdU detection kit (Beyotime) following the ‘manufacturer’s protocols. Briefly, cells were incubated in DMEM containing 20 μM EdU for 2 h. After fixing and permeabilizing as described above, cells were stained with Apollo and Hochest reagents in the dark. Images were taken by fluorescence microscopy and analyzed for EdU-positive cells using ImageJ.

### Glioma cell line xenografts and tumor formation in vivo

The animal experiments were approved by Xuzhou Medical University- Animal Center and the use committee. Normal Balb C (1-month-old males and females) were used as xenograft models. The glioma cell lines (U251) were implanted via injection of glioma cell line intracranially in the frontal cortex (3 mm below the brain, 2 mm rostral of the bregma, and 0.8 mm left midline) using a stereotactic device, and in the flank (subcutaneous) of normal Balb C mice. After implantation, the cells were allowed to grow inside the mouse brain for approximately 2 months. The experimental xenograft mice (males and female) were also treated with 5 µl concentrated testosterone via direct injection at the implanted area (subcutaneous xenograft) and through the tail blood vessel (brain xenograft) twice a week during the first month and every day during the last week before sacrifice. The control group xenograft males were castrated to avoid testosterone synthesis by the testis. Also, female mice were included as a control group due to the low expression of testosterone in females. The control group mice did not receive any testosterone treatment. We also proceeded with GDNF gene depletion by injecting 1 µl of adeno-associated virus vector for the brain xenograft and flank (subcutaneous xenograft).

### Immunohistochemistry

Tumor tissues were isolated from the mice after they were sacrificed. The tissues were immediately fixed in 4% paraformaldehyde for 24 h and embedded in paraffin. The embedded sections were sliced into 5 μm sections for staining. The deparaffinized and rehydrated sections were heated in citrate buffer at 121 °C for 30 min to retrieve antigen epitopes. The sections were incubated with 0.3% hydrogen peroxide (PV-9001, Origene) in methanol for 30 min to inhibit endogenous peroxidase activity. After non‐specific reactions had been blocked with 10% normal bovine serum, the sections were incubated with GDNF and GFAP primary antibody at 4 °C for 2 h. Then, the sections were washed with PBS and incubated with DAB (ZLI-9018, Origene) secondary antibody at 37 °C for 2 h. The stained sections were imaged under an inverted phase-contrast microscope.

### Hematoxylin–eosin (H-E)

The isolated tumor was fixed in formaldehyde, and obtained paraffin sections of 5 µm tumor tissue were stained for 2 min with hematoxylin solution, followed by 5 s of eosin staining and rapid acid-alcohol differential. Next, stepwise dehydration in increasing concentrations of alcohol was done, followed by immersion in xylene and mounting for microscopy. The images were captured under an Olympus confocal microscope, and the inflammatory cells were quantified using ImageJ.

### Statistical analysis

All values are expressed as mean ± SD. Statistical analyses were performed by Student’s *t* test for comparison between two groups or one-way ANOVA for multiple comparisons. *P* values < 0.05 were considered significant. *p < 0.05, **p < 0.01, ***p < 0.001, ****p < 0.0001, and ns indicates not significant. Data were processed and transformed using the EdgeR:log2(CPM + c) function of the online tool iDEP.96 (iDEP.96 (sdstate.edu)). Differential expression analysis was performed using the DESeq2 function of iDEP.96, where the expression profiles of GDNF overexpressed cells and controls were compared to identify the differential expression genes (DEGs). Adjusted *p* values were calculated using *t* tests. Genes from each sample with the following criteria were retained: (1) a log_2_ FC > 1 and (2) an adjusted *p* < 0.05. GraphPad Prism 8.0 software was used for the statistical analyses. “*n*” indicates the number of experimental replicates.

## Results

### Establishment of the relationship between testosterone, GDNF, and pro-inflammatory genes in U251 and HA

**Fig. 1:**
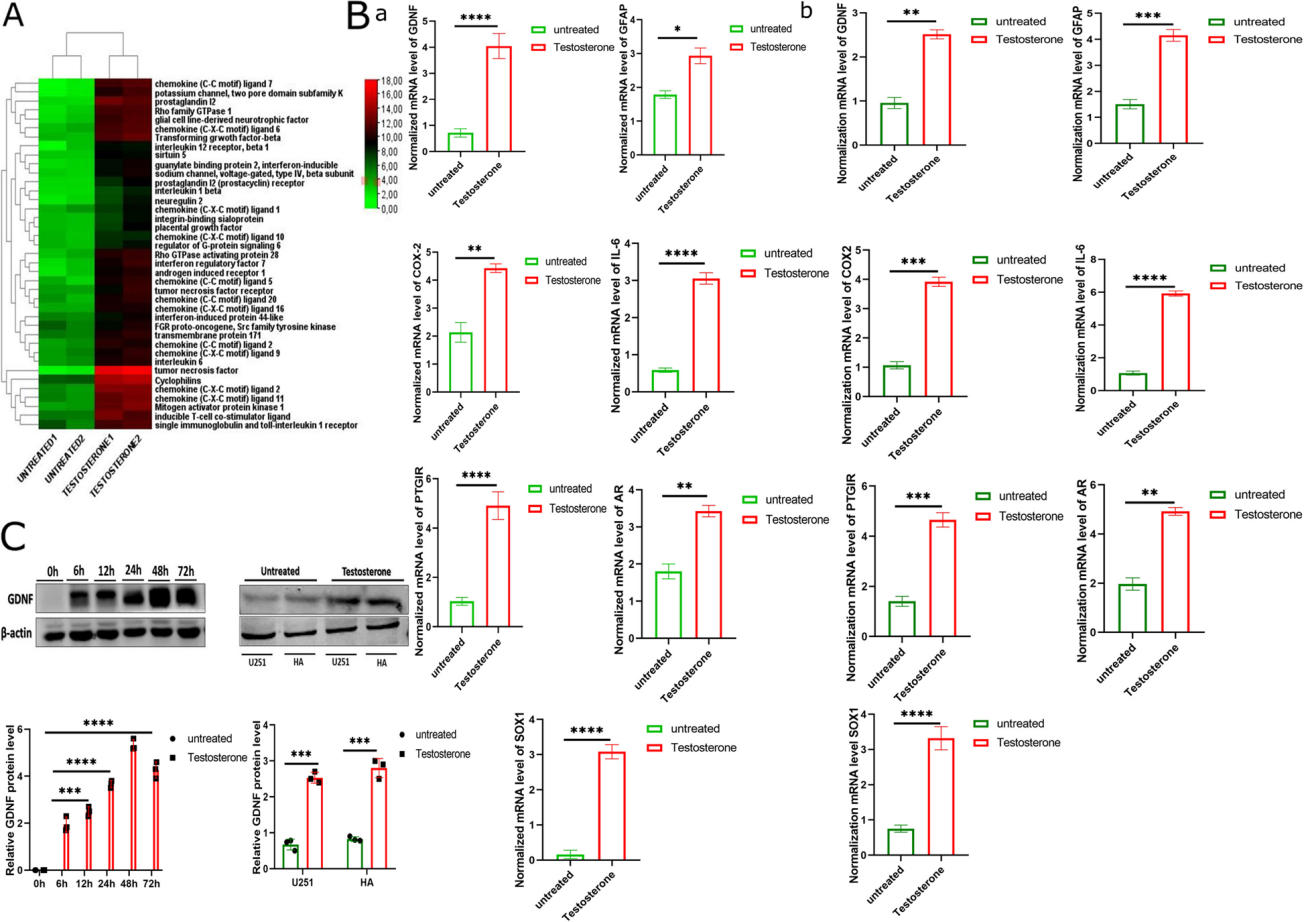
Differential gene expression between the control (untreated) and testosterone-treated U251 and HA. **A** Next-generation sequencing showing differential gene expression between the untreated group and testosterone-treated U251 (*n* = 2). We selected the genes that are involved in neuroinflammation only. **B** Reverse transcription-quantitative polymerization chain reaction (RT-qPCR) comparing GDNF*,* SOX1, IL-6, COX2, PTGIR, AR, and GFAP between the control (untreated) and testosterone-treated U251 and HA, **a** for U251 and **b** for HA (*n* = 3). **C** Immunoblotting comparing GDNF protein levels between the control (untreated) and testosterone-treated U251 and HA (*n* = 3). *p < 0.05, **p < 0.01, ***p < 0.001, and ****p < 0.0001

Glioma cell line (U251) and human astrocytes (HA) DNA sequencing through next-generation sequence showed that GDNF, chemokine, and pro-inflammatory cytokine expression significantly increased in the testosterone-treated group compared with the untreated group (Fig. 1A and Figure S1A). The PCR results showed that testosterone-treated U251 and HA had higher mRNA levels of GDNF, SOX-1, prostaglandin I2 (prostacyclin) receptor (PTGIR), androgen receptor (AR), interleukin-6 (IL-6), cyclooxygenase-2 (COX-2), and glial fibrillary acid protein (GFAP) than untreated U251 and HA (Fig. 1B). Our investigation showed that in the U251and HA treated with testosterone, GDNF protein increased significantly compared with the untreated groups (Fig. 1C). We also investigated the effect of testosterone treatment on U87, LN229, U251, and HA. GDNF protein level was highly increased in the testosterone-treated group compared with the untreated groups (Figure S1B). These results indicated that testosterone upregulation significantly upregulates GDNF, a pro-inflammatory cytokine, and their receptors in U251 and HA at the mRNA level.

### Testosterone promotes glioma cell line U251 and human astrocyte (HA) proliferation and invasion

**Fig. 2:**
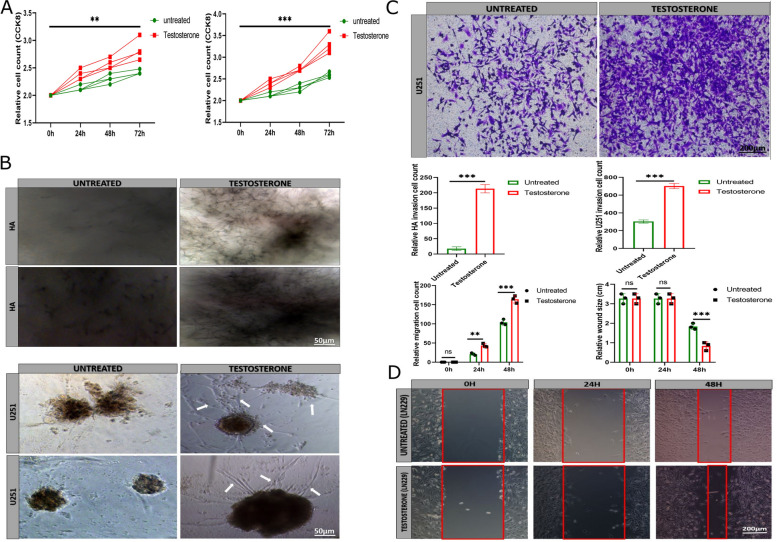
The effect of testosterone treatment on U251 and HA proliferation, invasion, and migration. **A** Cell counting kit 8 indicating U251 and HA survival and proliferation (*n* = 4). **B** Invasion assay comparing the untreated and testosterone-treated U251 and HA invasion on agarose gel (*n* = 3). The white arrow indicates invasive U251. **C** Transwell matrigel assay comparing the untreated and testosterone-treated U251 invasion and migration (*n* = 3). **D** wound-healing assay comparing the untreated and testosterone-treated LN229 migration (*n* = 3). **p < 0.01, ***p < 0.001, and ****p < 0.0001

The investigation of U251 and HA proliferation showed that the glioma cell lines treated with testosterone proliferated faster than the untreated control group (Fig. 2A). Invasion assay showed that testosterone causes spheroid U251 and HA invasion compared with the control group spheroid (Fig. 2B). These results were supported by the transwell matrigel assay (Fig. 2C, Figure S1C). The analysis of U251 migration also showed that testosterone contributes to glioma cell line migration compared with the untreated U251 groups (Fig. 2D, Figure S1D). Together, these results indicated that testosterone promotes U251 and HA proliferation, invasion, and U251 migration.

### Testosterone upregulates cyclophilin A protein level to enhance neuroprotection

**Fig. 3:**
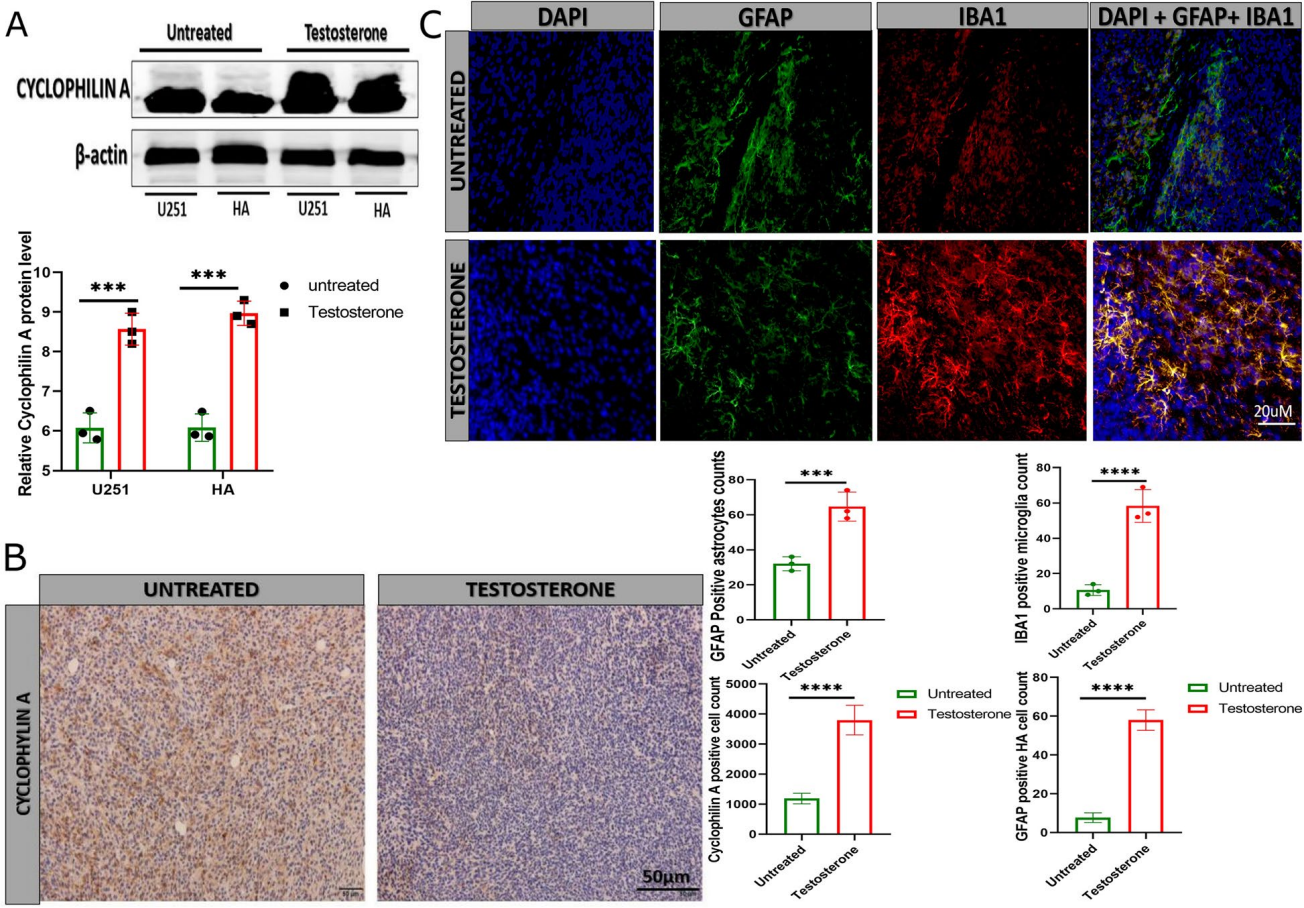
Testosterone upregulates cyclophilin A expression in vitro and in vivo. **A** Immunoblotting detection of cyclophilin **A** protein level in U251 and HA (*n* = 3). **B** Immunohistochemistry comparing cyclophilin A-positive cells between the untreated and testosterone-treated xenograft tissue (*n* = 3). **C** Immunofluorescence comparing GFAP (green) and IBA1 (red) positive inflammatory astrocyte between the untreated and testosterone-treated brain tissue (*n* = 3). GFAP (green) indicated the total astrocyte and IBA1 of the inflammatory microglia. ***p < 0.001, and ****p < 0.0001

It is well known that GDNF expression significantly increases during inflammation to protect the brain cells against the adverse effects of inflammation and apoptosis [14, 21]. Several studies also reported that GDNF regulates brain cells' survival and improves their functioning in Parkinson's disease therapy. Another protein upregulated in the testosterone-treated group, cyclophilin A, was reported to have a neuroprotective effect during inflammation [22, 23]. The immunoblotting result showed that cyclophilin A protein level was high in testosterone-treated U251 and HA compared with the untreated U251 and HA (Fig. 3A). The same result was observed in LN229, U87, U251, and HA (Figure S1E). Immunohistochemistry analysis of tumor tissue showed a high level of cyclophilin A positive cells in testosterone-treated tumor tissue compared with the untreated tumor tissue (Fig. 3B). The immunofluorescence using GFAP and IBA1 showed a high number of inflammatory astrocytes in testosterone-treated mice compared with the untreated mice tissue (Fig. 3C). These results suggested that testosterone controls cyclophilin A upregulation in U251 and HA. Combining testosterone treatment and inflammation-induced GDNF and cyclophilin A upregulation in U251 and HA might scaffold their survival exponentially.

### Testosterone contributes to U251 and HA survival via inflammation maintenance and GDNF upregulation

**Fig. 4:**
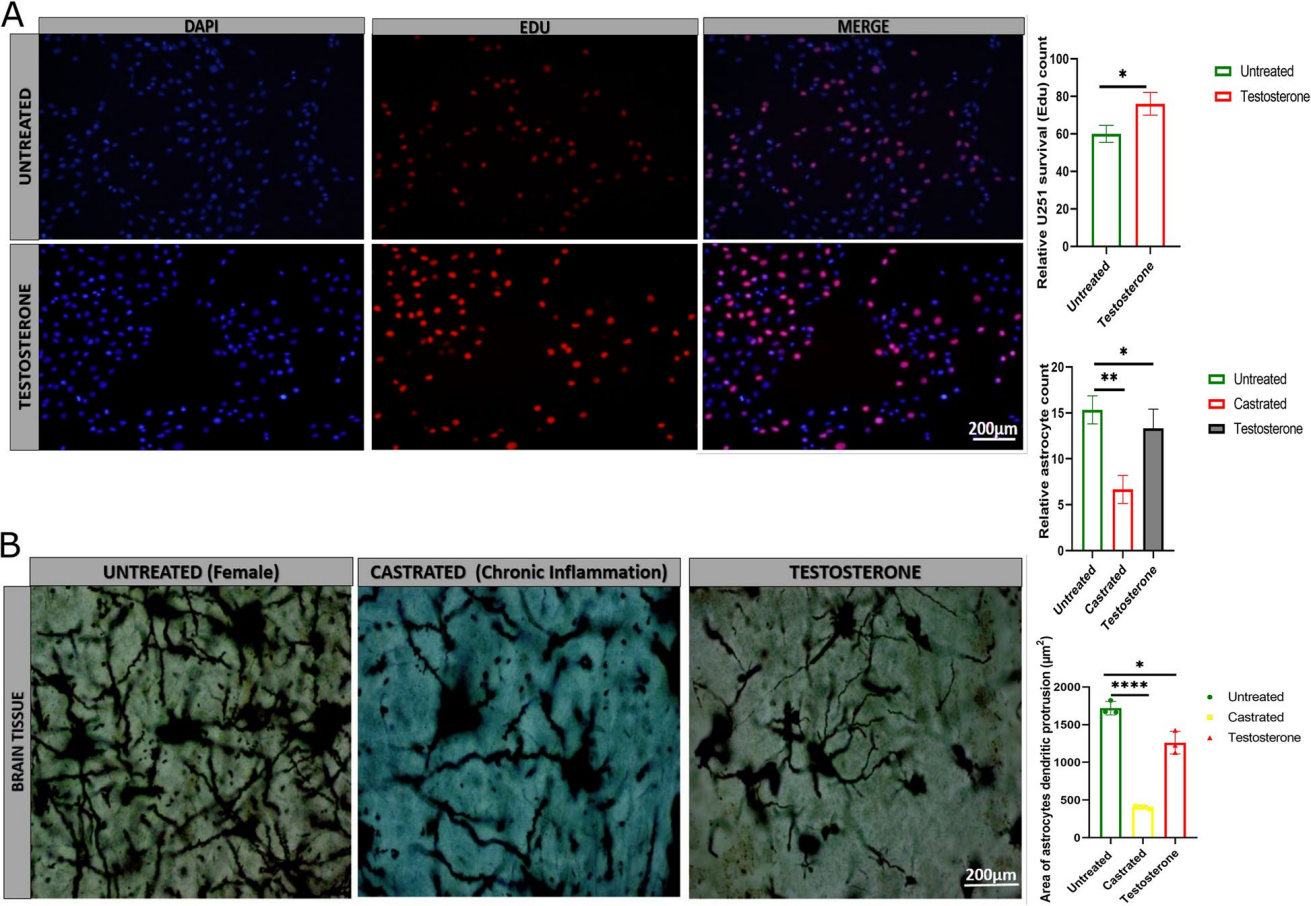
Testosterone contributes to U251 and HA survival. **A** 5-Ethynyl-2’-Deoxyuridine (EDU) staining comparing U251 survival between the untreated and testosterone-treated groups (*n* = 3). **B** Comparison of brain astrocyte shape (protrusions) between the untreated and testosterone-treated brain tissue in inflammation condition using Sholl analysis (*n* = 3). In the normal female mouse tissue, the astrocytes' shape looked good, and the axons and dendritic protrusions were well differentiated. In the castrated mouse tissue, the astrocyte shape and axons are affected (altered and discontinuous axon protrusions) due to tumor inflammation and a decrease in testosterone level. In testosterone-treated castrated mice, we can observe the regeneration of axon protrusions and improvement of the astrocyte shape even under inflammation. *p < 0.05 and **p < 0.01

The brain cells' survival is critical for the brain's electrical, hormonal, psychological, and movement coordination. The death of brain cells causes the imbalance and dysfunction of the central nervous system resulting in loss or distortion in nervous impulses, hormonal secretion, and organ functioning. Edu analysis of U251 indicated that testosterone significantly improved U251 survival compared with the untreated groups (Fig. 4A, Figure S1F). The staining of mice brain cortex showed that cortex astrocyte shapes (protrusions) were highly damaged in castrated and testosterone-treated mice than in the control group female mice astrocytes (Fig. 4B). Also, we observed alterations in astrocyte axons (proximal and distal processes) protrusions caused by inflammation in the testosterone-treated group. The testosterone-treated mice astrocytes showed axon and dendritic protrusion regeneration compared with the castrated mice astrocytes (Figure S1G). These results indicated that testosterone regulated U251 and HA survival, and rescued astrocytes shape to regain function.

### Testosterone upregulation in the tumor microenvironment acts as a barrier that transforms macrophages that cross the barrier into tumor-associated macrophages

**Fig. 5:**
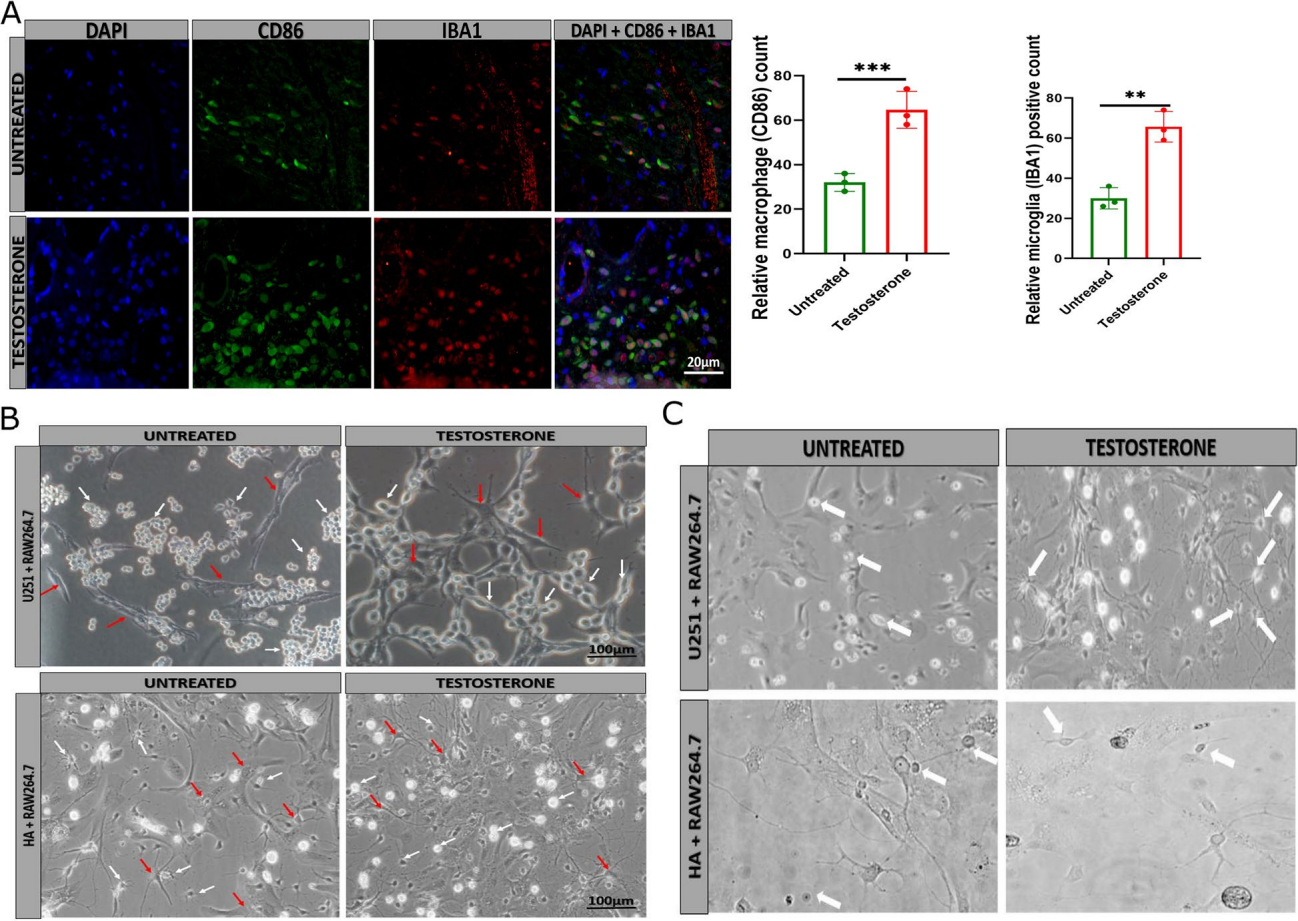
Testosterone promotes pro-inflammatory macrophage recruitment and activation. **A** Immunofluorescence comparing pro-inflammatory macrophages (CD86) and microglial (Iba1) between the untreated and testosterone-treated tumor tissue (*n* = 3) **B** Contrast image showing macrophage (Raw264.7) interaction with U251 and HA between the untreated and testosterone-treated groups (*n* = 4). The white arrow indicates the macrophages and the red arrow indicates U251 or HA. **C** Contrast image comparing macrophage activation between the untreated and testosterone-treated groups (*n* = 4). The activated microphage have outgrowth while the non-activated macrophages did not have. **p < 0.01 and ***p < 0.001

The immunofluorescence analysis of tumor tissue revealed that macrophages within the tumor express pro-inflammatory macrophage markers CD86 in testosterone-treated mice, but untreated mice display few pro-inflammatory macrophages (Fig. 5A). A similar result was observed in female mice (Figure S1H), and a few pro-inflammatory were detected in the untreated female mice; however, the number of pro-inflammatory macrophages in the testosterone-treated female increased. Iba1-positive cells represent the total microglia in tumor tissue. These results suggested that testosterone via the oncogenes and cytokines highly contributes to macrophage polarization. Further investigation showed physical interaction between the U251, astrocytes, and macrophages (Fig. 5B, Figure S1I). The contrast images showed that testosterone activates macrophages while the untreated group macrophages are not activated (Fig. 5C). These results indicated that testosterone treatment increases pro-inflammatory macrophage recruitment into the brain tumor tissue compared with the untreated group tumor tissue. The cytokines and chemokines secretion by the U251 and HA glioma cell line (next-generation sequencing) contribute to macrophage (pro-inflammatory phenotype) recruitments. The comparison of the control groups between male and female mice revealed that a few (negligible) pro-inflammatory macrophages in females compared with males.

### Testosterone treatment regulates NRF2 and ERK1/2, crucial for cytokine and chemokine secretion

**Fig. 6:**
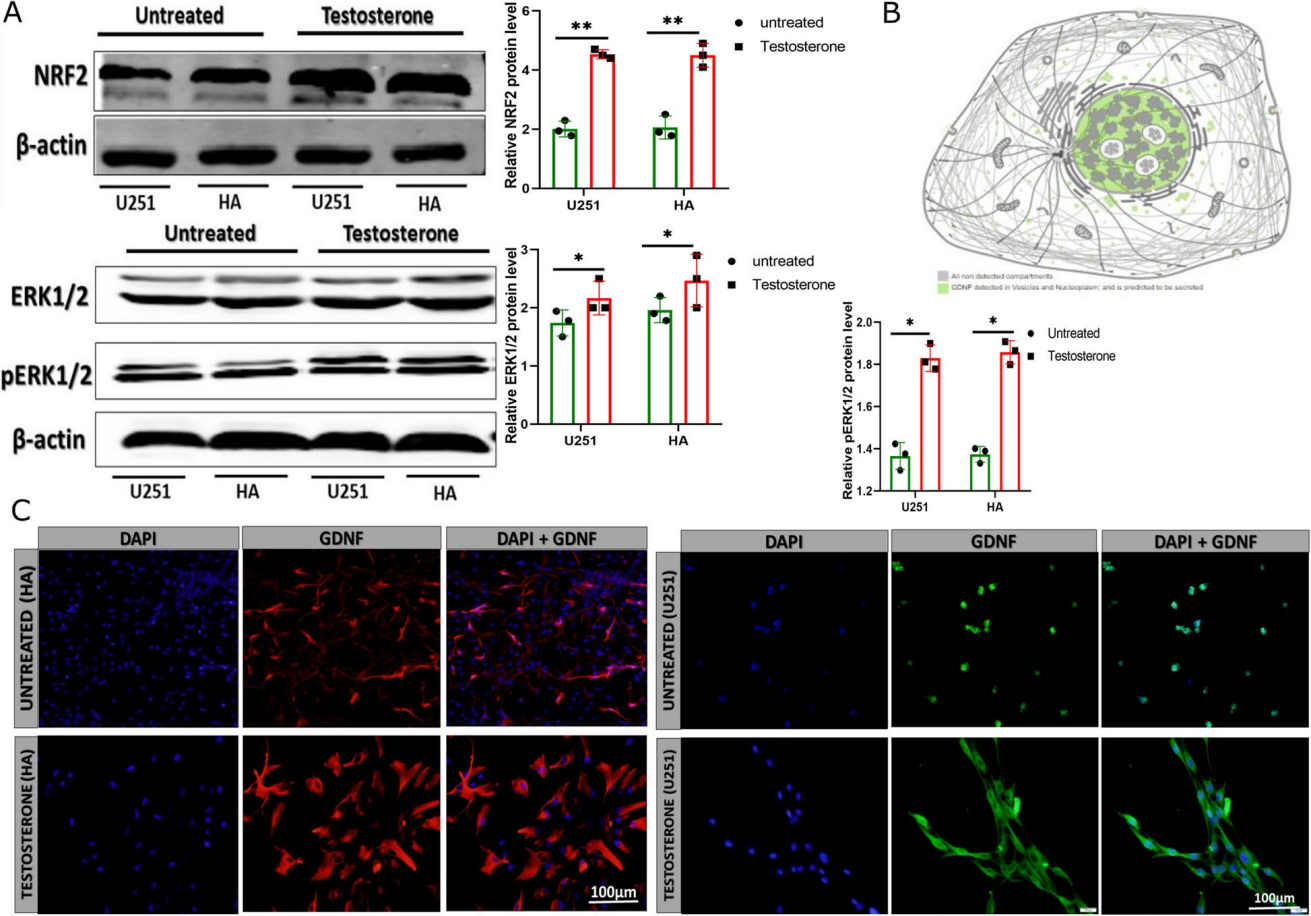
The testosterone upregulates pro-inflammatory genes NRF2, ERK1/2, and pERK1/2 protein level and GDNF translocation. **A** Immunoblotting detection of NRF2, ERK1/2, and pERK1/2 protein levels in U251 and HA (*n* = 3). **B** Protein atlas indicating GDNF subcellular location. **C** Immunofluorescence detection of GDNF subcellular localization in human astrocyte (red) and U251 (green) (*n* = 4). *p < 0.05 and **p < 0.01

The analysis of differential gene expression by next-generation sequence showed that sirtuin 5 (SIRT5), extracellular signal-regulated kinase (ERK ½), phosphorylated extracellular signal-regulated kinase (pERK ½), and nuclear factor erythroid 2-related factor 2 (NRF2) are also highly expressed. The western blot results showed that ERK1/2 and NRF2 expressions are effectively upregulated in testosterone-treated U251 and HA compared with their respective untreated group (Fig. 6A, Figure S1J). The immunofluorescence analysis revealed that the GDNF subcellular location changed. The protein atlas data showed that GDNF is highly found in the glial cell nucleus (Fig. 6B); however, our investigation showed that GDNF could translocate. In the testosterone-treated U251 and HA, GDNF was found in both cytoplasm (highly expressed) and nucleus (modestly expressed). At the same time, in the control group of U251 and HA, it was observed to be substantially expressed in the nuclei and perinuclear surroundings (Fig. 6C, Figure S1K). The immunostaining of tumor tissue indicated a high level of GDNF and NRF2-positive cells in testosterone-treated tumor tissue compared with their respective control groups, which showed low GDNF and NRF2-positive cells. The hematoxylin–eosin staining revealed that the level of inflammatory cells was higher in testosterone-treated tumor tissue than in the control group tumor tissue (Figure S1L).

### GDNF knockout drastically reduced pro-inflammatory gene expression and microglial survival

**Fig. 7:**
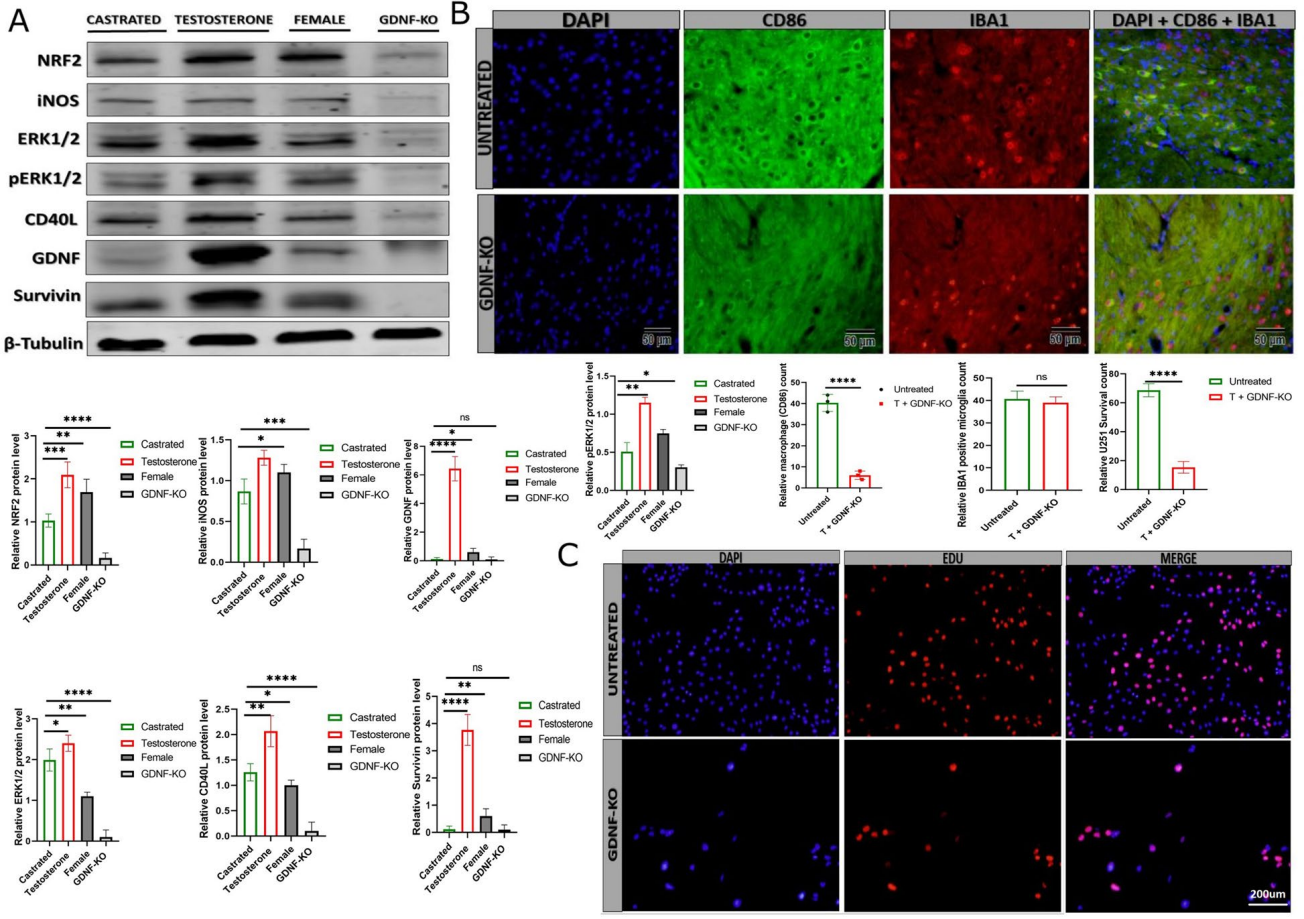
The effect of GDNF knockout on inflammatory protein level, pro-inflammatory macrophage recruitment, and microglial survival. **A** Western blot detection of NRF2, iNOS, ERK1/2, pERK1/2, CD40L, GDNF, and Survivin in castrated, testosterone, female, and GDNF knockout (*n* = 3).** B** Immunofluorescence detection of pro-inflammatory macrophages (CD86) and microglia (Iba1) between the untreated and GDNF knockout + testosterone-treated brain tissue, Dapi was used to stain U251 nuclei (*n* = 3). **C** Edu staining comparing the untreated and GDNF knockout + testosterone treated U251 survival (*n* = 3). Dapi was used to stain U251 nuclei. *p < 0.05, **p < 0.01, ***p < 0.001, ****p < 0.0001

To determine the role of GDNF on the immune response to inflammation and glioma cell survival in vivo, we knock down the GDNF gene and proceed to pro-inflammatory protein detection via western blot. The western blot results indicated that NRF2, iNOS, ERK1/2, pERK1/2, and CD40L were low in GDNF knockout (GDNF-KO) compared with their respective protein levels in testosterone-treated, castrated, and female mice (Fig. 7A). The highest expression was detected in testosterone-treated mouse tissue. The immunostaining of the brain tissues to detect pro-inflammatory macrophages showed that the number of pro-inflammatory macrophages drastically decreased (Fig. 7B). The analysis of microglial survival via the Edu assay indicated that the GDNF knockout in microglial negatively impacts its survival compared with the untreated microglial, even in the testosterone-enrich environment (Fig. 7C, Figure S1M). These results indicate that GDNF is critical for pro-inflammatory cytokine expression and contributes to activated pro-inflammatory macrophage recruitment.

## Discussion

The serum concentration of testosterone in male mice is between 300 and 1000 ng/dl (3–10 ng/ml), and in females, 15 to 70 ng/dl (0.15–0.7 ng/ml). Several studies reported that testosterone regulates dopamine levels (and vice versa) [24, 25] to ensure brain cells' survival [26]. The amplification of prostaglandin and androgen receptors indicated that testosterone binds to its receptor on the U251 and HA plasma membrane to activate signal transduction, leading to the upregulation of genes. Oncogenes stimulation and cytokines secretion in testosterone-treated U251 and HA are associated with the neuroprotective effect of testosterone induced by GDNF and cyclophilin A protein upregulation, contributing to U251 and HA proliferation. This proliferation is also due to dampened apoptosis, as the Edu assay indicates. Several studies have reported the role of sex differences in brain cancer [27–29]; although Juyeun Lee et al. 2022 report on testosterone functions in tumor suppression, the authors did not demonstrate their hypothesis through investigations in the laboratory. Testosterone regulates macrophage recruitment and GDNF upregulation to protect the brain cells and microglia against apoptosis without significantly affecting the inflammation state due to macrophage polarization and tumor hallmark that significantly reduces cancer cell phagocytosis [30]. The anti-inflammatory function of testosterone is attenuated by the cytokines and oncogenes secreted by cancer cells and pro-inflammatory macrophages to favor damaged microglia cells’ (Fig. 4B) survival and proliferation via GDNF upregulation necessary for tumor growth.

Immune cells permitting tumor growth are indicative of pathological conditions. Physiologically, immune cells elicit anti-tumor responses and adaptively modulate their inflammatory responses to limit the negative effects on brain cell damage. However, our observation indicated that instead of fighting the cancer cells, the macrophages in the tumor microenvironment were biasedly polarized towards pro-inflammatory phenotype. During the early stage of inflammation, macrophage activations facilitate adaptive functions such as impeding abnormal cell proliferation via phagocytosis or the synthesis of cytokines to eliminate unwanted cells [31, 32]. Conversely, in the tumor microenvironment, these mechanisms enhance the proliferative cells’ survival and adaptation to the immune response, enabling them to evade immunosurveillance and ultimately scaffolding cancer progression [33, 34]. Intriguingly, testosterone facilitates the aforementioned pathological cascade by stimulating the upregulation of oncogenes and cytokines, which enhances interactions between cancer cells and macrophages in the tumor microenvironment. Due to the neuroprotection conferred by GDNF in central nervous system cancers [14], its upregulation in testosterone-treated glioma cell lines and mice tissue protects glioma cell lines against immune response. Additionally, as a member of the transforming growth factor beta (TGF-β) superfamily, GDNF can regulate and reprogramme macrophages into tumor-associated macrophages (TAM) in the tumor microenvironment characterized by hypersecretion of chemokines [35, 36] as shown in the differential gene expression (Fig. 1A). The upregulation of cyclophilin A is highly expressed during inflammation and contributes to proteins transformation from cis-to-trans—thereby altering protein functions [37–39]. As such, it is speculated that cyclophilin A could induce the transformation of cytokines and chemokines and the inactivation of tumor suppressor genes even when they are expressed. The upregulation of toll-like receptor 2 (TLR-2) on testosterone-treated glioma cell lines increases their recognition by innate immune cells as normal cells [40]. The upregulation of TLR-2 on the glioma cell membrane facilitates cell proliferation because the innate immune cells did not attack (phagocytosis) the glioma cells [41]. Synergistically, these mechanisms contribute to cancer cell proliferation and tumor growth and progression.

The loss of axons is caused by neuroinflammation at the earlier stage of inflammation, the transformation of the brain neuron-like astrocytes, and that transformation significantly affects the astrocyte's shape, function, and behavior [42, 43]. The aberrant astrocyte survival brought on by GDNF overexpression [14] and the hormonal instability that results from the inability to control electrical impulses in the central nervous system accelerates the onset and progression of the disease [9]. Under normal conditions, the upregulation of testosterone activates androgen receptors in the hypothalamus to modulate its levels [44]. We bypassed this regulatory system of the organism by injecting the testosterone directly into the tumor area or microenvironment.

The upregulation of SIRT5, ERK1/2, and NRF2 clearly indicates neuroinflammation activity caused by testosterone treatment. It is documented that SIRT5 is highly expressed in the cells experiencing mitochondrial stress and autophagy, both of which promote inflammation [45, 46]. ERK1/2 and NRF2 also contribute to brain cell and microglia inflammation and regulate immune cell recruitment in inflammatory areas [47–49]. NRF2 is also a transcription factor that regulates antioxidant and cellular protective genes [50, 51]. Testosterone induces the upregulation of all these genes to contribute to glioma cell line survival and proliferation. The tumor microenvironment changes are caused by the high synthesis of chemokines and cytokines produced by glioma cell lines and immune cells in the tumor microenvironment, which consequently stimulates tumor growth via cell recruitment and proliferation. Our data suggest that testosterone is a crucial hormone that regulates the central nervous system diseases such as brain and spinal cancers by activating oncogenes, pro-inflammatory cytokines, and chemokine synthesis that facilitates astrocyte inflammation, cancer cell proliferation, and tumor progression. GDNF and cyclophilin A upregulation contributed substantially to cancer cell survival and adaptation against immune responses and surveillance [14, 52]. The protein atlas analysis indicated that GDNF, SOX1, cyclophilin-A, NRF2, ERK1/2, COX2, and IL-6 are highly detected in the thalamus and hypothalamus, whose activity relies on testosterone (Figure S2). A further investigation into the epigenetics modifications and metabolic contribution to GDNF upregulation induced by testosterone will provide insight and help advance cancer therapy development. Also, identifying the GDNF translocation mechanism and its contribution to U251 and HA survival and neuroinflammation can help reduce glioma cell proliferation and tumor growth, as GDNF proteins are known to regulate brain cell survival.

## Conclusion

Together, our results indicated that testosterone regulates GDNF and cytokine upregulation in glioma. Treating U251 and HA with testosterone modified their function and behavior (proliferation, invasion, and migration). The secretion of cytokines by U251 and the recruitment of pro-inflammatory macrophages contribute to inflammation maintenance necessary for glioma cells and astrocytes survival [9, 14] and tumor growth [13]. Males expressing more testosterone than females may explain the higher mortality rate in males with glioblastoma compared to females.

### Supplementary Information

**Additional file 1: Figure S1A.** Next-generation sequencing of human astrocytes (U251). The treatment of U251 with testosterone upregulates the cytokines and oncogenes compared with the untreated groups. Next-generation sequencing of human astrocytes (HA). The treatment of HA with testosterone upregulates the cytokines and oncogenes compared with the untreated groups. **Figure S1B.** Western blot detection of GDNF in human astrocyte (HA), U251, LN229, and U87: The treatment of HA, U251, LN229, and U87 with testosterone significantly increased GDNF protein level compared with the untreated groups. **p<0.01 and ****p<0.0001. **Figure S1C.** Transwell assay indicating LN229 glioma cell line invasion. The results indicated that the LN229 glioma cell line invasion in the untreated LN229 was slightly lower than in testosterone-treated LN229 in which the invasion ability highly increased. *p<0.05. **Figure S1D.** Wound-healing assay indicating U251 migration abilities: the results showed that the U251 migration ability is high testosterone-treated U251 compared to the untreated U251 in which the U251 glioma cell line migration was low. **p<0.01 and ***p<0.001. **Figure S1E.** Western blot detection of Cyclophilin A in U251, LN229, U87, and HA. The results showed that the cyclophilin A protein level in the untreated glioma cell line was lower than in testosterone-treated glioma cell lines in which cyclophilin A protein level significantly increased. ***p<0.001. **Figure S1F.** LN229 glioma cell line survival test via EDU assay. The staining of the LN229 glioma cell lines with EDU solution showed that the testosterone-treated LN229 glioma cell lines survival significantly increased compared with the untreated LN229 glioma cell lines. *p<0.05. **Figure S1G.** Brain tissue staining with trypan blue to reveal astrocyte shape. The observation of astrocyte shape showed that the untreated mice brain has a better astrocyte shape followed by testosterone-treated astrocytes. The castrated mice astrocyte axons were completely gone; however, the castrated group treated with testosterone showed a regeneration of axons. ***p<0.001, and ****p<0.0001. **Figure S1H.** Immunofluorescence detection of pro-inflammatory macrophages in female mice brain. the control group showed few CD86-positive macrophages; however, CD68-positive macrophages were detected. In testosterone-treated female mice, we detected both CD86 and CD68 macrophages significantly. *p<0.05 and **p<0.01. **Figure S1I.** Co-culture showing interactions of LN229 with Raw264.7 in the untreated and testosterone treated conditions. **Figure S1J.** Western blot detection of NRF2 in U251, LN229, U87, and HA. The results showed that the NRF2 protein level in the untreated glioma cell line was lower than in testosterone-treated glioma cell lines in which the NRF2 protein level significantly increased. * ***p<0.001. Western blot detection of ERK1/2 and pERK1/2 in U251, LN229, U87, and HA. The results showed that the ERK1/2 and pERK1/2 protein levels in the untreated glioma cell line was lower than in testosterone-treated glioma cell lines in which ERK1/2 and pERK1/2 protein levels significantly increased. the highest difference between the untreated and testosterone-treated pERK1/2 was observed in U251 and HA cells. *p<0.05, **p<0.01, ***p<0.001, and ****p<0.0001. **Figure S1K.** Immunofluorescence showing GDNF subcellular localization in human astrocyte (HA). The results showed that GDNF was found in the untreated group HA nucleus, while in testosterone treated group, GDNF is found mainly in the cytoplasm.Immunofluorescence showing GDNF subcellular localization in U251. The results showed that GDNF was found in the untreated group U251 nucleus, while in testosterone treated group, GDNF is found mainly in the cytoplasm. Immunofluorescence showing GDNF subcellular localization in LN229. The results showed that GDNF was found in the untreated group LN229 nucleus, while in testosterone treated group, GDNF is found mainly in the cytoplasm. **Figure S1L.** Hematoxylin-eosin (H-E) of tumor tissue comparing inflammatory cell between the control (untreated) and testosterone treated groups. The inflammatory cell is higher in testosterone treated tissue than the untreated tissue. ***P<0.001. **Figure S1M.** LN229 glioma cell line survival test via EDU assay. The staining of the LN229 glioma cell line with EDU solution showed that the Untreated LN229 glioma cell lines survival significantly increased compared with the GDNF-KO LN229 glioma cell lines, which showed a low survival. ****p<0.0001.

**Additional file 2: Figure S2.** The protein atlas analysis indicated that GDNF, SOX1, cyclophilin-A, NRF2, ERK1/2, COX2, and IL-6 are highly detected in the thalamus and hypothalamus, whose activity relies on testosterone.

## Data Availability

The datasets used and/or analyzed during the current study are available from the corresponding author on reasonable request.

## Abbreviations

GDNF: Glial cell line-derived neurotrophic factor
SOX-1: Sex-determining region Y-box1
ERK1/2: Extracellular signal-regulated kinase 1/2
NRF2: Nuclear factor erythroid 2-related factor 2
SIRT5: Sirtuin 5
AR: Androgen receptor
PTGIR: Prostaglandin I2 (prostacyclin) receptor
AR: Androgen receptor
IL-6: Interleukin-6
COX-2: Cyclooxygenase-2
GFAP: Glial fibrillary acid protein
iNOS: Inducible nitric acid synthase

## References

[1] Carrano A, Juarez JJ, Incontri D, Ibarra A, Guerrero Cazares H. Sex-specific differences in glioblastoma. Cells. 2021;10(7). 10.3390/cells10071783. 10.3390/cells10071783 PMC8303471 34359952

[2] Ostrom QT, Kinnersley B, Wrensch MR, Eckel-Passow JE, Armstrong G, Rice T (2018). Sex-specific glioma genome-wide association study identifies new risk locus at 3p21.31 in females, and finds sex-differences in risk at 8q24.21. Sci Rep..

[3] Vining B, Ming Z, Bagheri-Fam S, Harley V. Diverse regulation but conserved function: SOX9 in vertebrate sex determination. Genes (Basel). 2021;12(4). 10.3390/genes12040486. 10.3390/genes12040486 PMC8066042 33810596

[4] Jakob S, Lovell-Badge R (2011). Sex determination and the control of Sox9 expression in mammals. FEBS J.

[5] Garcia I, Aldaregia J, MarjanovicVicentic J, Aldaz P, Moreno-Cugnon L, Torres-Bayona S (2017). Oncogenic activity of SOX1 in glioblastoma. Sci Rep.

[6] Lin YW, Tsao CM, Yu PN, Shih YL, Lin CH, Yan MD (2013). SOX1 suppresses cell growth and invasion in cervical cancer. Gynecol Oncol.

[7] Munkley J, Lafferty NP, Kalna G, Robson CN, Leung HY, Rajan P (2015). Androgen-regulation of the protein tyrosine phosphatase PTPRR activates ERK1/2 signalling in prostate cancer cells. BMC Cancer.

[8] McHenry J, Carrier N, Hull E, Kabbaj M (2014). Sex differences in anxiety and depression: role of testosterone. Front Neuroendocrinol.

[9] Kanwore K, Guo XX, Abdulrahman AA, Kambey PA, Nadeem I, Gao D (2021). SOX1 is a backup gene for brain neurons and glioma stem cell protection and proliferation. Mol Neurobiol.

[10] Lin LF, Doherty DH, Lile JD, Bektesh S, Collins F (1993). GDNF: a glial cell line-derived neurotrophic factor for midbrain dopaminergic neurons. Science.

[11] Blennerhassett MG, Lourenssen SR (2022). Obligatory activation of SRC and JNK by GDNF for survival and axonal outgrowth of postnatal intestinal neurons. Cell Mol Neurobiol.

[12] Chen G, Du Y, Li X, Kambey PA, Wang L, Xia Y (2021). Lower GDNF serum level is a possible risk factor for constipation in patients with Parkinson disease: a case-control study. Front Neurol.

[13] Wang M, Han X, Zha W, Wang X, Liu L, Li Z (2022). GDNF promotes astrocyte abnormal proliferation and migration through the GFRalpha1/RET/MAPK/pCREB/LOXL2 signaling axis. Mol Neurobiol.

[14] Ayanlaja AA, Zhang B, Ji G, Gao Y, Wang J, Kanwore K (2018). The reversible effects of glial cell line-derived neurotrophic factor (GDNF) in the human brain. Semin Cancer Biol.

[15] Rodriguez-Lozano DC, Pina-Medina AG, Hansberg-Pastor V, Bello-Alvarez C, Camacho-Arroyo I (2019). Testosterone promotes glioblastoma cell proliferation, migration, and invasion through androgen receptor activation. Front Endocrinol (Lausanne).

[16] Zaker H, Razi M, Mahmoudian A, Soltanalinejad F (2022). Boosting effect of testosterone on GDNF expression in Sertoli cell line (TM4); comparison between TM3 cells-produced and exogenous testosterone. Gene.

[17] Davey RA, Grossmann M (2016). Androgen receptor structure, function and biology: from bench to bedside. Clin Biochem Rev.

[18] Wellberg EA, Checkley LA, Giles ED, Johnson SJ, Oljira R, Wahdan-Alaswad R (2017). The androgen receptor supports tumor progression after the loss of ovarian function in a preclinical model of obesity and breast cancer. Horm Cancer.

[19] Capper CP, Rae JM, Auchus RJ (2016). The metabolism, analysis, and targeting of steroid hormones in breast and prostate cancer. Horm Cancer.

[20] Xiong Y, Liu L, Zhu S, Zhang B, Qin Y, Yao R (2017). Precursor N-cadherin mediates glial cell line-derived neurotrophic factor-promoted human malignant glioma. Oncotarget.

[21] Morel L, Domingues O, Zimmer J, Michel T. Revisiting the Role of Neurotrophic Factors in Inflammation. Cells. 2020;9(4). 10.3390/cells9040865. 10.3390/cells9040865 PMC7226825 32252363

[22] Gegunde S, Alfonso A, Alvarino R, Alonso E, Botana LM (2021). Cyclophilins A, B, and C role in human T lymphocytes upon inflammatory conditions. Front Immunol.

[23] Daneri-Becerra C, Valeiras B, Gallo LI, Lagadari M, Galigniana MD. Cyclophilin A is a mitochondrial factor that forms complexes with p23 - correlative evidence for an anti-apoptotic action. J Cell Sci. 2021;134(3). 10.1242/jcs.253401. 10.1242/jcs.253401 33361281

[24] Purves-Tyson TD, Owens SJ, Double KL, Desai R, Handelsman DJ, Weickert CS (2014). Testosterone induces molecular changes in dopamine signaling pathway molecules in the adolescent male rat nigrostriatal pathway. PLoS ONE.

[25] Purves-Tyson TD, Handelsman DJ, Double KL, Owens SJ, Bustamante S, Weickert CS (2012). Testosterone regulation of sex steroid-related mRNAs and dopamine-related mRNAs in adolescent male rat substantia nigra. BMC Neurosci.

[26] Ohira K (2020). Dopamine as a growth differentiation factor in the mammalian brain. Neural Regen Res.

[27] Claus EB, Cannataro VL, Gaffney SG, Townsend JP (2022). Environmental and sex-specific molecular signatures of glioma causation. Neuro Oncol.

[28] Turaga SM, Silver DJ, Bayik D, Paouri E, Peng S, Lauko A (2020). JAM-A functions as a female microglial tumor suppressor in glioblastoma. Neuro Oncol.

[29] Lee J, Silver D, Chung Y-M, Sharifi N, Lathia J (2022). IMMU-24. Testosterone functions as a tumor suppressor in glioblastoma. Neuro-Oncology..

[30] Kuntzel T, Bagnard D. Manipulating macrophage/microglia polarization to treat glioblastoma or multiple sclerosis. Pharmaceutics. 2022;14(2). 10.3390/pharmaceutics14020344. 10.3390/pharmaceutics14020344 PMC8877500 35214076

[31] Ren Y, Khan FA, Pandupuspitasari NS, Zhang S (2017). Immune evasion strategies of pathogens in macrophages: the potential for limiting pathogen transmission. Curr Issues Mol Biol.

[32] Malaguarnera L. Influence of resveratrol on the immune response. Nutrients. 2019;11(5). 10.3390/nu11050946. 10.3390/nu11050946 PMC6566902 31035454

[33] Raman D, Baugher PJ, Thu YM, Richmond A (2007). Role of chemokines in tumor growth. Cancer Lett.

[34] Hao NB, Lu MH, Fan YH, Cao YL, Zhang ZR, Yang SM (2012). Macrophages in tumor microenvironments and the progression of tumors. Clin Dev Immunol.

[35] Stuelten CH, Zhang YE (2021). Transforming growth factor-beta: an agent of change in the tumor microenvironment. Front Cell Dev Biol.

[36] Gratchev A (2017). TGF-beta signalling in tumour associated macrophages. Immunobiology.

[37] Nigro P, Satoh K, O'Dell MR, Soe NN, Cui Z, Mohan A (2011). Cyclophilin A is an inflammatory mediator that promotes atherosclerosis in apolipoprotein E-deficient mice. J Exp Med.

[38] Xue C, Sowden MP, Berk BC (2018). Extracellular and Intracellular Cyclophilin A, Native and post-translationally modified, show diverse and specific pathological roles in diseases. Arterioscler Thromb Vasc Biol.

[39] Chen ZJ, Vetter M, Chang GD, Liu S, Che D, Ding Y (2004). Cyclophilin A functions as an endogenous inhibitor for membrane-bound guanylate cyclase-A. Hypertension.

[40] Delneste Y, Beauvillain C, Jeannin P (2007). Innate immunity: structure and function of TLRs. Med Sci (Paris).

[41] Xun Y, Yang H, Kaminska B, You H (2021). Toll-like receptors and toll-like receptor-targeted immunotherapy against glioma. J Hematol Oncol.

[42] DiSabato DJ, Quan N, Godbout JP (2016). Neuroinflammation: the devil is in the details. J Neurochem..

[43] Siracusa R, Fusco R, Cuzzocrea S (2019). Astrocytes: role and functions in brain pathologies. Front Pharmacol.

[44] Iovino M, Messana T, Iovino E, De Pergola G, Guastamacchia E, Giagulli VA (2019). Neuroendocrine mechanisms involved in male sexual and emotional behavior. Endocr Metab Immune Disord Drug Targets.

[45] Bringman-Rodenbarger LR, Guo AH, Lyssiotis CA, Lombard DB (2018). Emerging Roles for SIRT5 in metabolism and Cancer. Antioxid Redox Signal.

[46] Jaiswal A, Xudong Z, Zhenyu J, Saretzki G (2022). Mitochondrial sirtuins in stem cells and cancer. FEBS J.

[47] Chen X, Wei G, Li D, Fan Y, Zeng Y, Qian Z (2022). Sirtuin 1 alleviates microglia-induced inflammation by modulating the PGC-1alpha/Nrf2 pathway after traumatic brain injury in male rats. Brain Res Bull.

[48] Mohan S, Gupta D (2018). Crosstalk of toll-like receptors signaling and Nrf2 pathway for regulation of inflammation. Biomed Pharmacother.

[49] Lailler C, Louandre C, Morisse MC, Lhossein T, Godin C, Lottin M, et al. ERK1/2 signaling regulates the immune microenvironment and macrophage recruitment in glioblastoma. Biosci Rep. 2019;39(9). 10.1042/BSR20191433. 10.1042/BSR20191433 PMC6744584 31467175

[50] Tonelli C, Chio IIC, Tuveson DA (2018). Transcriptional regulation by Nrf2. Antioxid Redox Signal.

[51] He F, Ru X, Wen T. NRF2, a transcription factor for stress response and beyond. Int J Mol Sci. 2020;21(13). 10.3390/ijms21134777. 10.3390/ijms21134777 PMC7369905 32640524

[52] Lee J (2010). Role of cyclophilin a during oncogenesis. Arch Pharm Res.

