# Supplementary material for: Testosterone upregulates glial cell line-derived neurotrophic factor (GDNF) and promotes neuroinflammation to enhance glioma cell survival and proliferation
Source: Inflamm Regen. 2023 Oct 13;43:49. doi: 10.1186/s41232-023-00300-7 (PMC10571473; doi:10.1186/s41232-023-00300-7)
Supplement: Supplementary file 1 — Additional file 1: Figure S1A. Next-generation sequencing of human astrocytes (U251). The treatment of U251 with testosterone upregulates the cytokines and oncogenes compared with the untreated groups. Next-generation sequencing of human astrocytes (HA). The treatment of HA with testosterone upregulates the cytokines and oncogenes compared with the untreated groups. Figure S1B. Western blot detection of GDNF in human astrocyte (HA), U251, LN229, and U87: The treatment of HA, U251, LN229, and U87 with testosterone significantly increased GDNF protein level compared with the untreated groups. **p<0.01 and ****p<0.0001. Figure S1C. Transwell assay indicating LN229 glioma cell line invasion. The results indicated that the LN229 glioma cell line invasion in the untreated LN229 was slightly lower than in testosterone-treated LN229 in which the invasion ability highly increased. *p<0.05. Figure S1D. Wound-healing assay indicating U251 migration abilities: the results showed that the U251 migration ability is high testosterone-treated U251 compared to the untreated U251 in which the U251 glioma cell line migration was low. **p<0.01 and ***p<0.001. Figure S1E. Western blot detection of Cyclophilin A in U251, LN229, U87, and HA. The results showed that the cyclophilin A protein level in the untreated glioma cell line was lower than in testosterone-treated glioma cell lines in which cyclophilin A protein level significantly increased. ***p<0.001. Figure S1F. LN229 glioma cell line survival test via EDU assay. The staining of the LN229 glioma cell lines with EDU solution showed that the testosterone-treated LN229 glioma cell lines survival significantly increased compared with the untreated LN229 glioma cell lines. *p<0.05. Figure S1G. Brain tissue staining with trypan blue to reveal astrocyte shape. The observation of astrocyte shape showed that the untreated mice brain has a better astrocyte shape followed by testosterone-treated astrocytes. The castrated mice astrocyte axons [file 41232_2023_300_MOESM1_ESM.docx]

**Supplementary figure S2 :**

**Fig. S1A :**


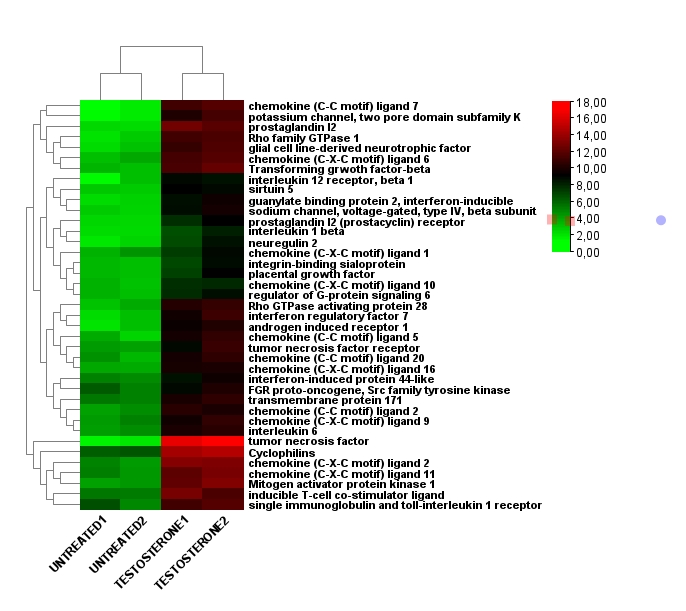


**Next-generation sequencing of human astrocytes (U251).** The treatment of U251 with testosterone upregulates the cytokines and oncogenes compared with the untreated groups.

**
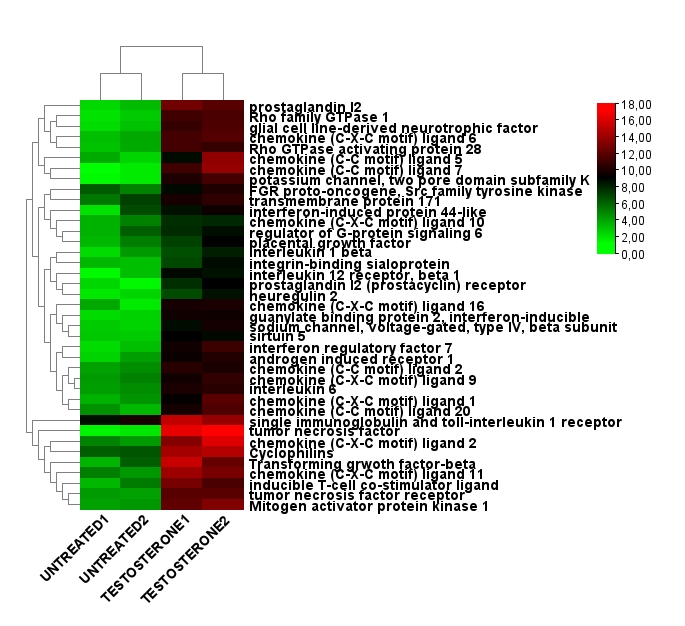
**

**Next-generation sequencing of human astrocytes (HA).** The treatment of HA with testosterone upregulates the cytokines and oncogenes compared with the untreated groups.

**Fig. S1B**:


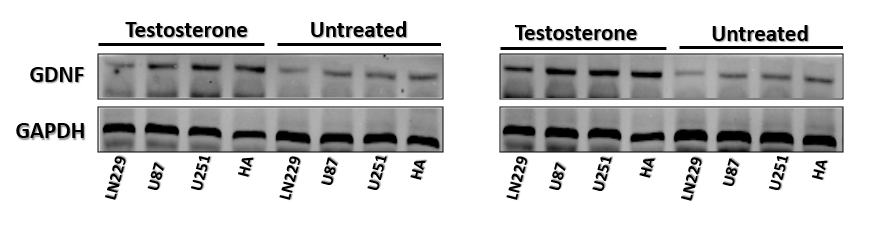


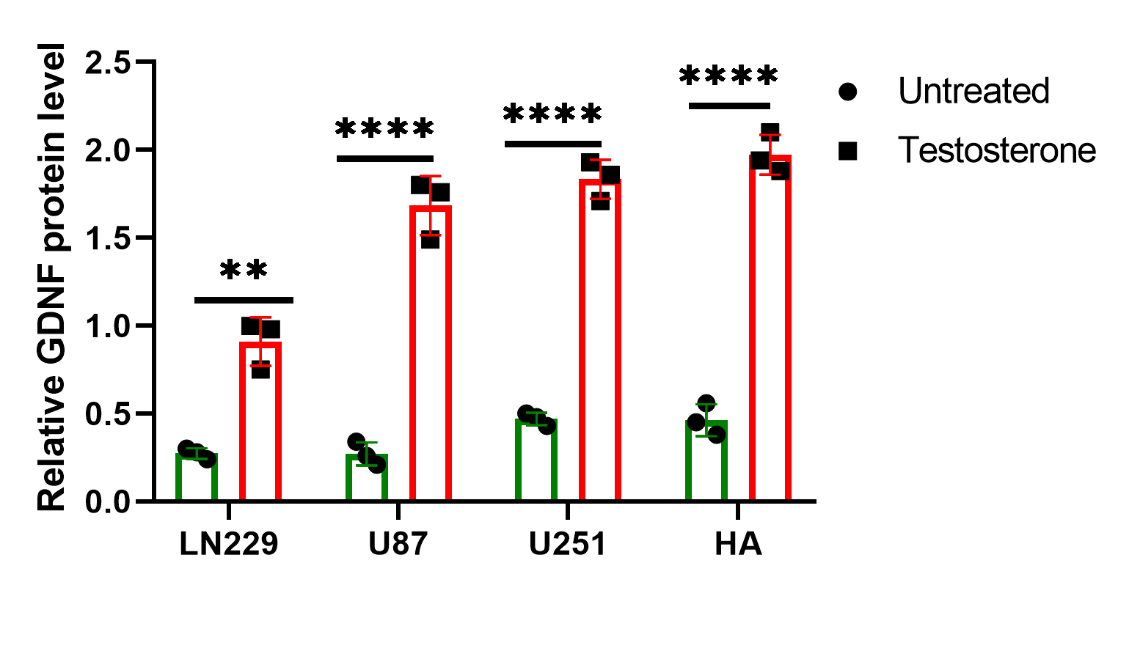


**Western blot detection of GDNF in human astrocyte (HA), U251, LN229, and U87:** The treatment of HA, U251, LN229, and U87 with testosterone significantly increased GDNF protein level compared with the untreated groups. ******p<0.01 and ********p<0.0001.

**Fig. S1C**:


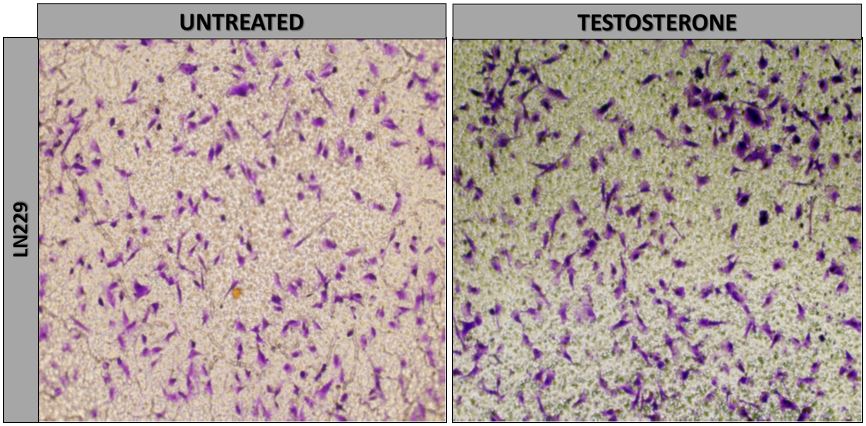


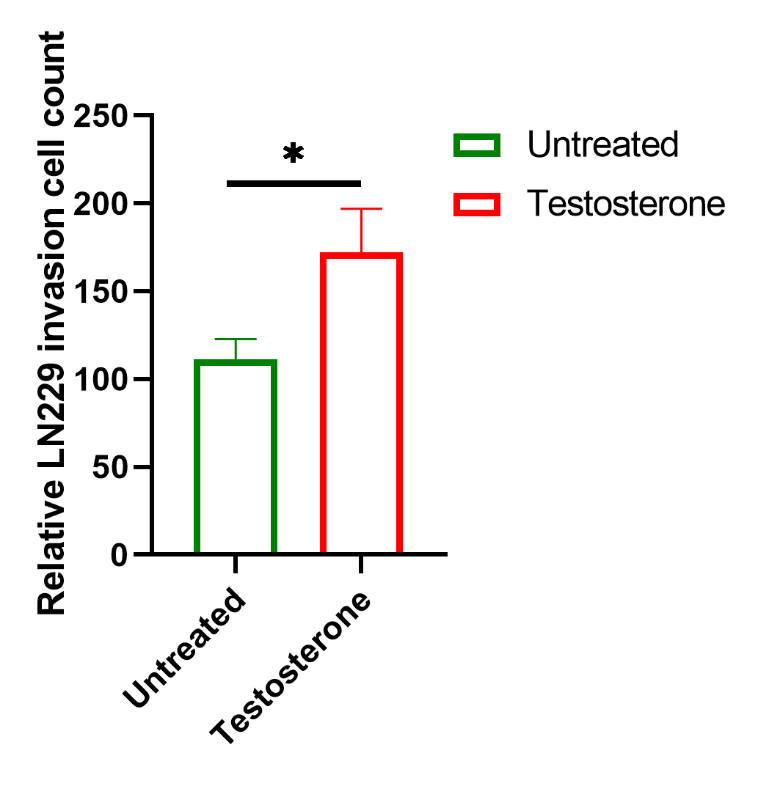


**Transwell assay indicating LN229 glioma cell line invasion**. The results indicated that the LN229 glioma cell line invasion in the untreated LN229 was slightly lower than in testosterone-treated LN229 in which the invasion ability highly increased. *****p<0.05.

**Fig. S1D**:


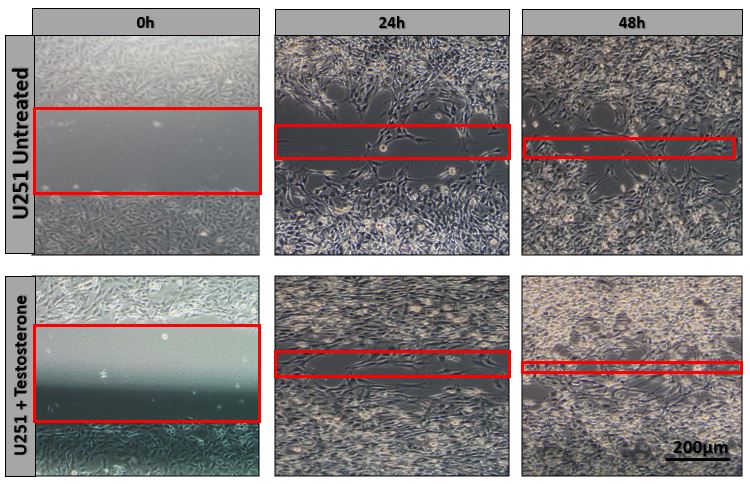


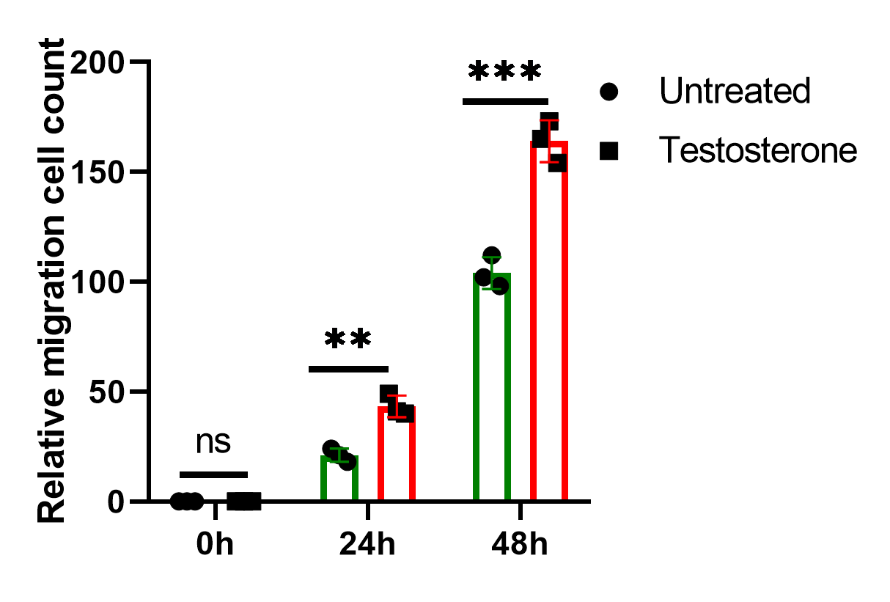


**Wound-healing assay indicating U251 migration abilities**: the results showed that the U251 migration ability is high testosterone-treated U251 compared to the untreated U251 in which the U251 glioma cell line migration was low. ******p<0.01 and *******p<0.001.

**Fig S1E:**


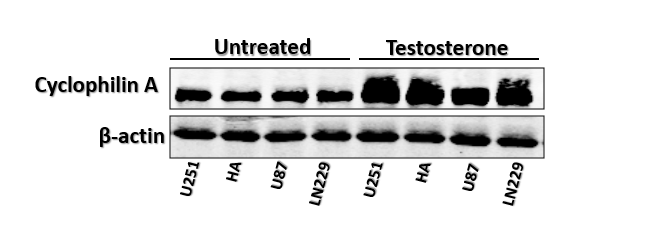

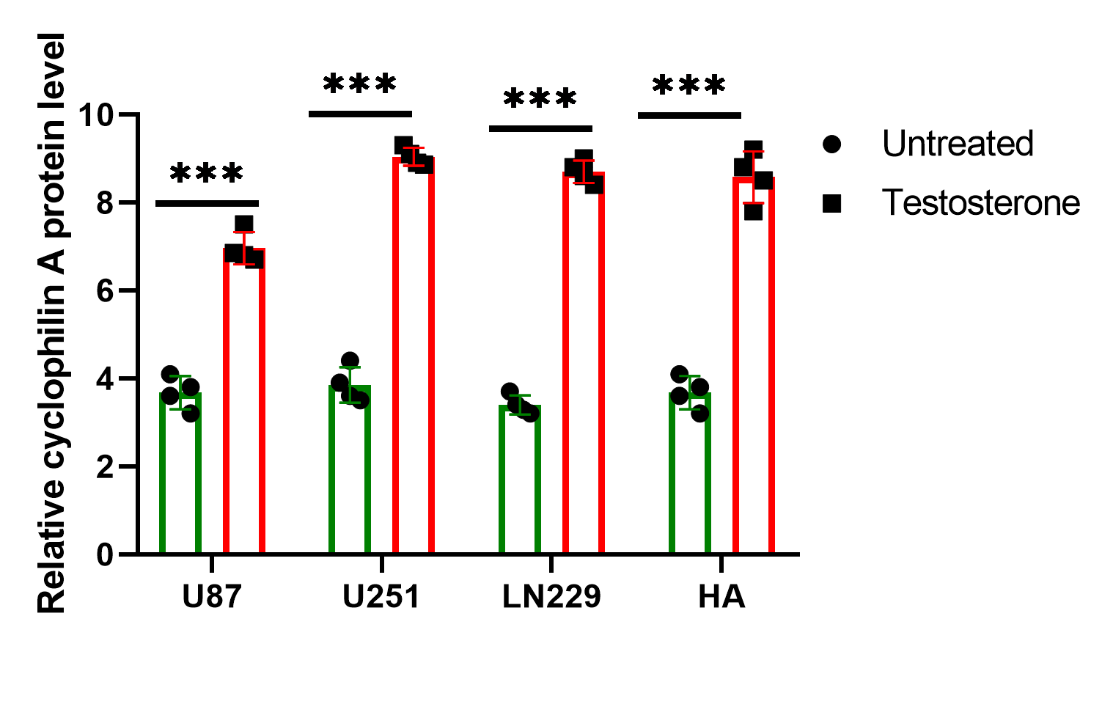


Western blot detection of Cyclophilin A in U251, LN229, U87, and HA. The results showed that the cyclophilin A protein level in the untreated glioma cell line was lower than in testosterone-treated glioma cell lines in which cyclophilin A protein level significantly increased. *******p<0.001.

**Fig S1F:**


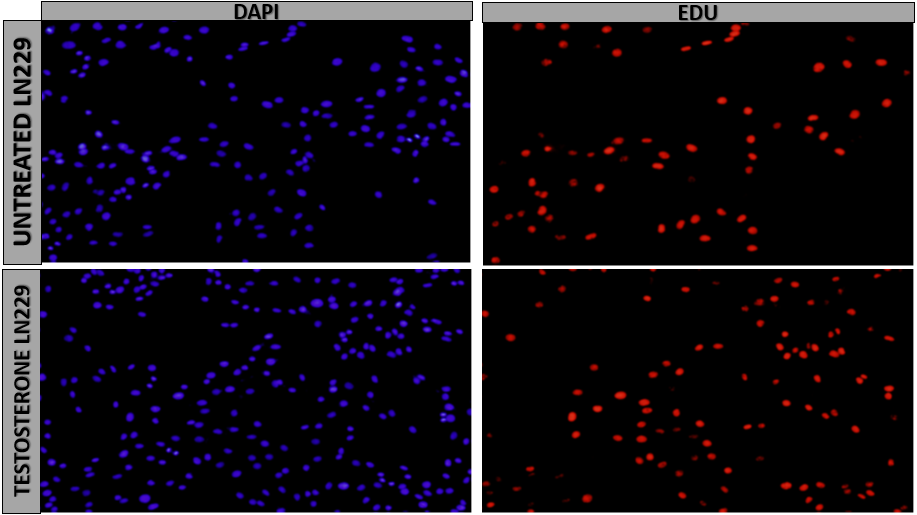


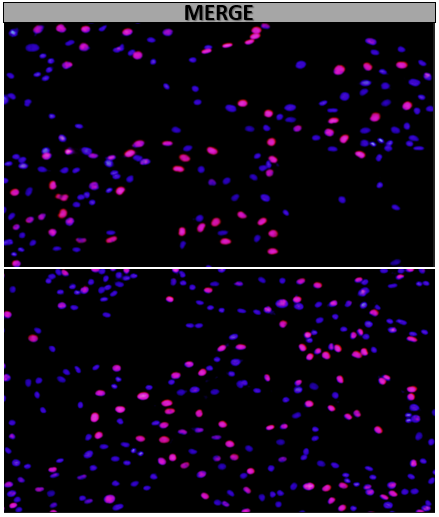

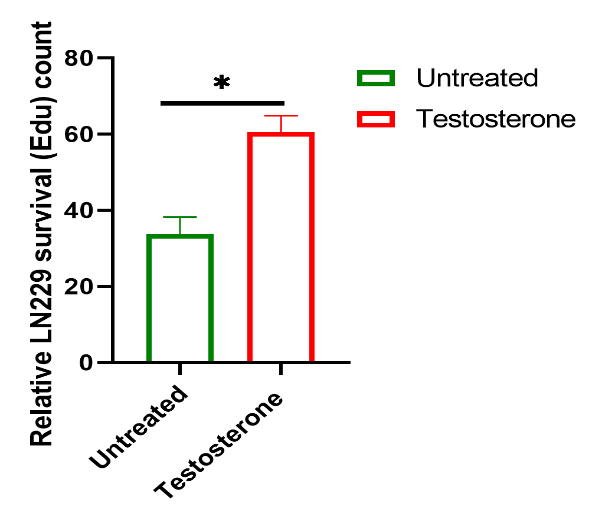


LN229 glioma cell line survival test via EDU assay. The staining of the LN229 glioma cell lines with EDU solution showed that the testosterone-treated LN229 glioma cell lines survival significantly increased compared with the untreated LN229 glioma cell lines. *****p<0.05.

**Fig. S1G:**


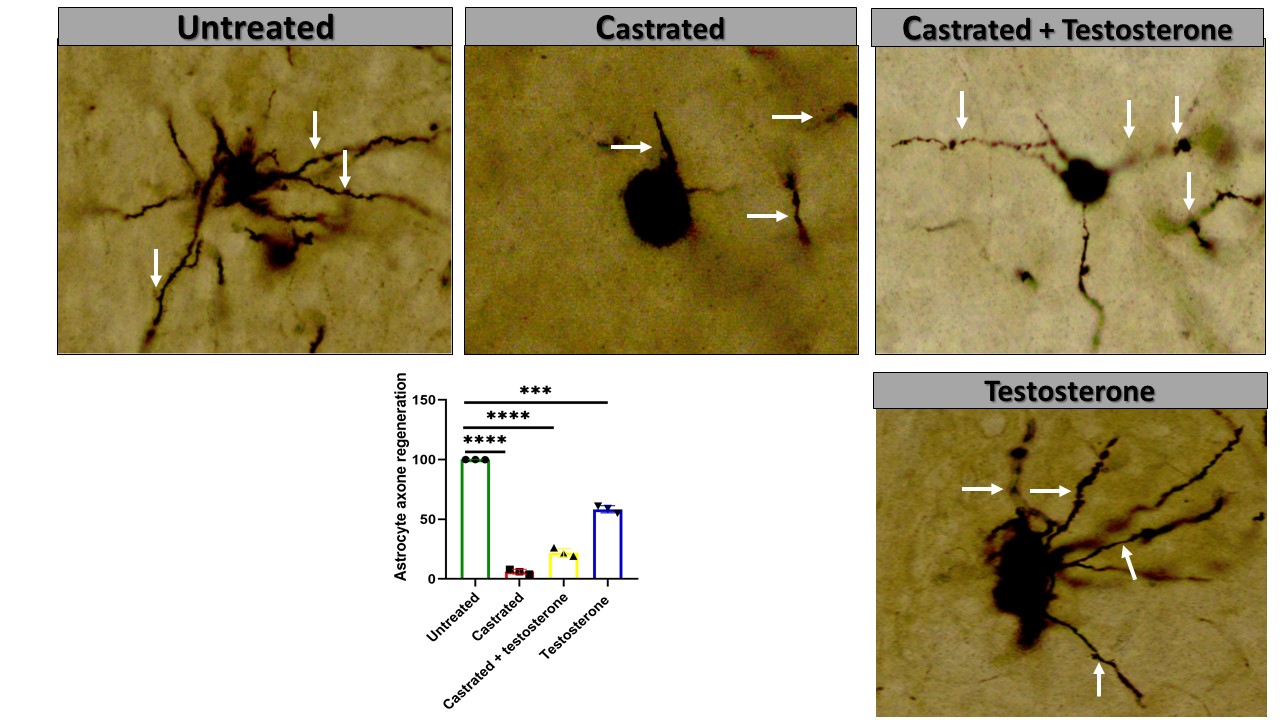


Brain tissue staining with trypan blue to reveal astrocyte shape. The observation of astrocyte shape showed that the untreated mice brain has a better astrocyte shape followed by testosterone-treated astrocytes. The castrated mice astrocyte axons were completely gone; however, the castrated group treated with testosterone showed a regeneration of axons. *******p<0.001, and ********p<0.0001.

**Fig. S1H:**


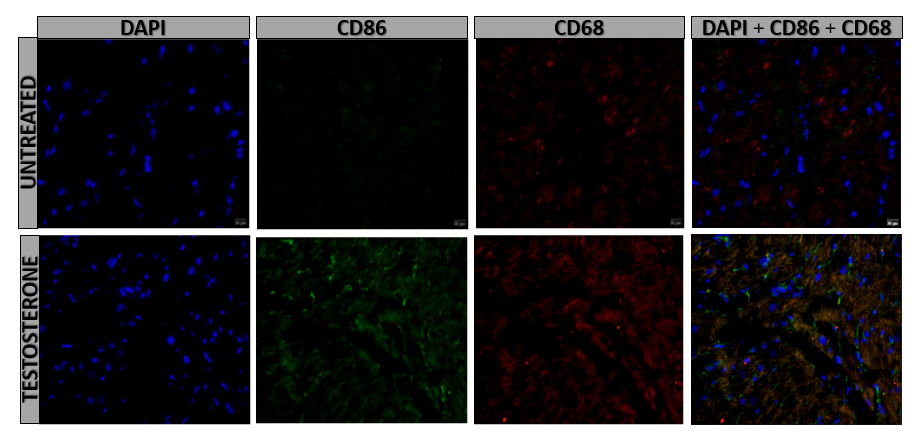


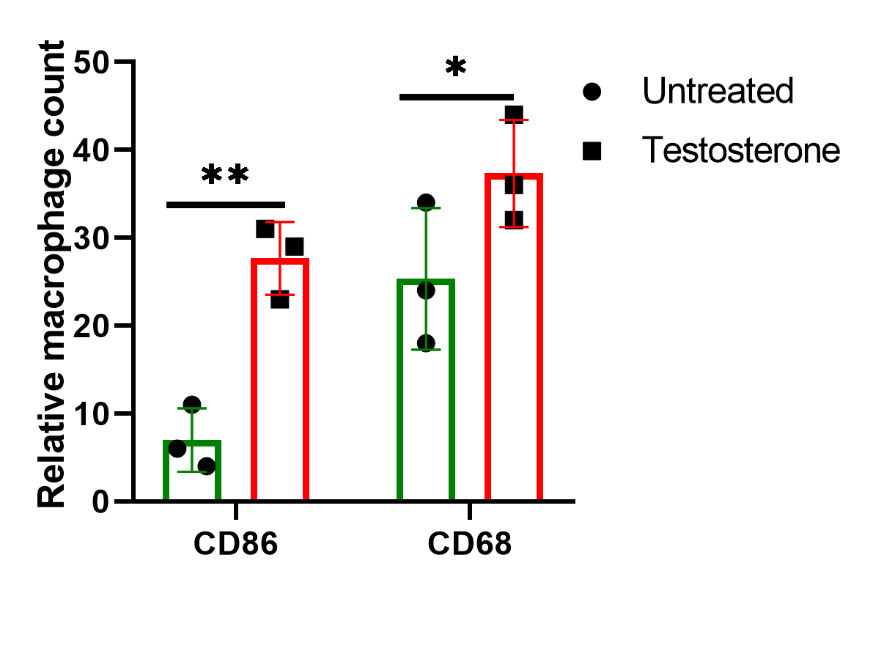


**Immunofluorescence detection of pro-inflammatory macrophages in female mice brain**. the control group showed few CD86-positive macrophages; however, CD68-positive macrophages were detected. In testosterone-treated female mice, we detected both CD86 and CD68 macrophages significantly. *****p<0.05 and ******p<0.01.

**Fig. S1I**:


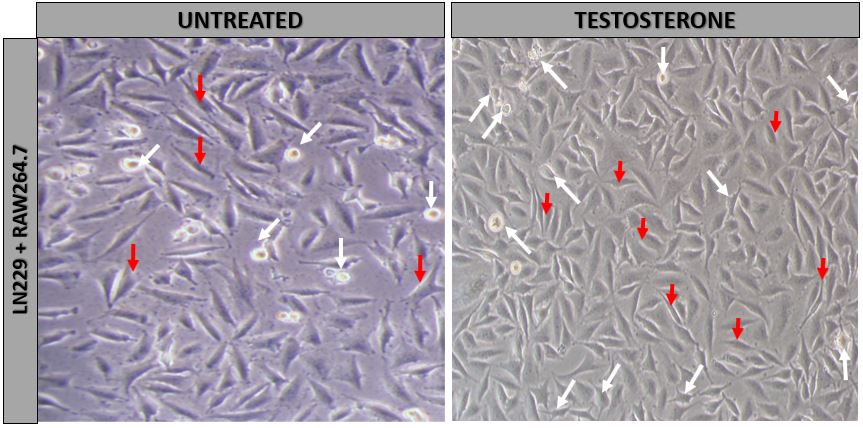


Co-culture showing interactions of LN229 with Raw264.7 in the untreated and testosterone treated conditions.

**Fig. S1J:**


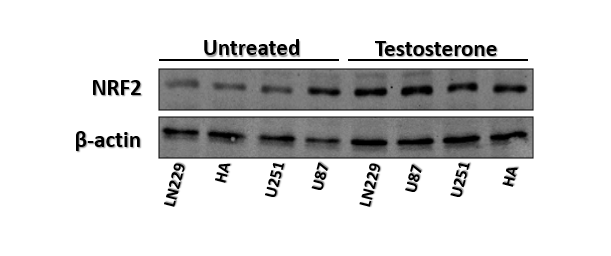

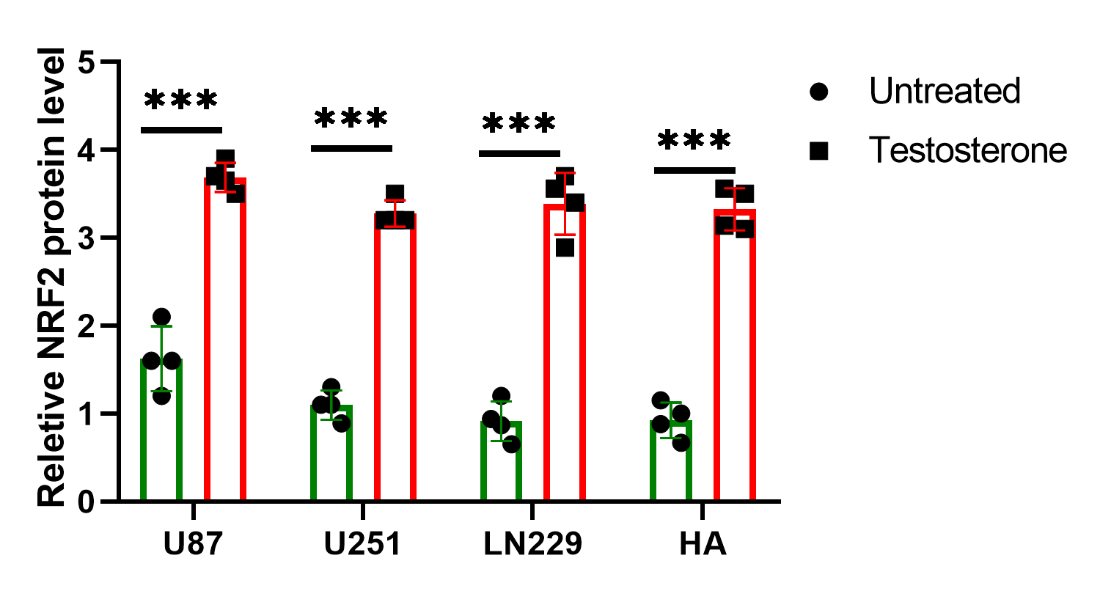


Western blot detection of NRF2 in U251, LN229, U87, and HA. The results showed that the NRF2 protein level in the untreated glioma cell line was lower than in testosterone-treated glioma cell lines in which the NRF2 protein level significantly increased. ***** *******p<0.001.


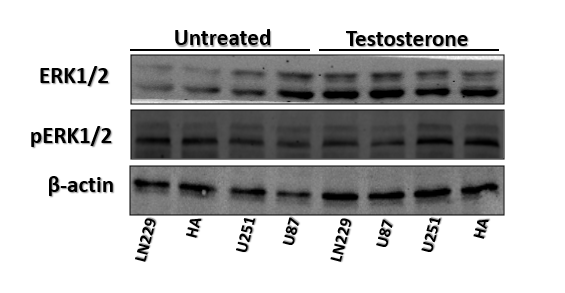

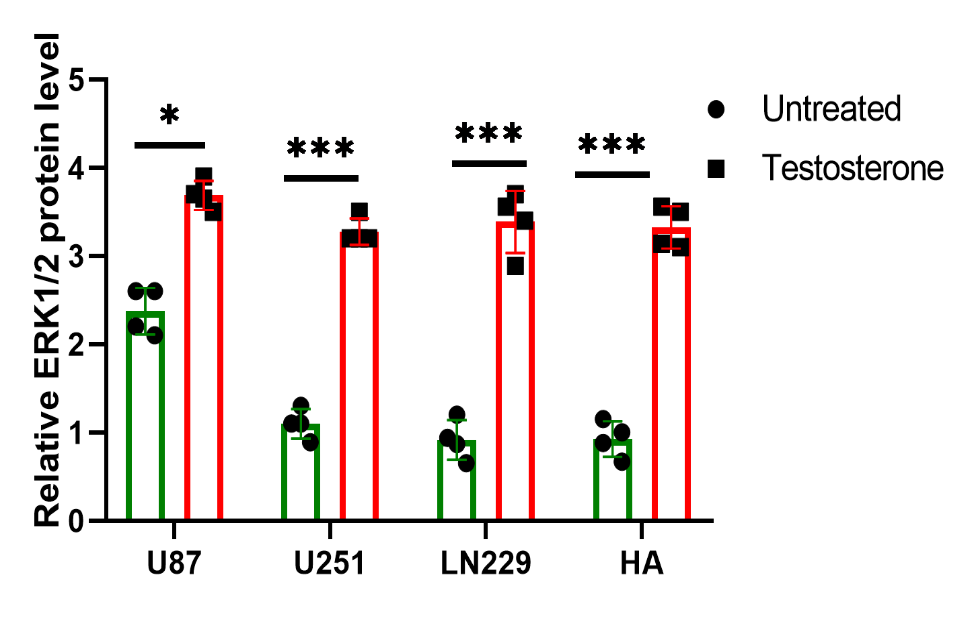

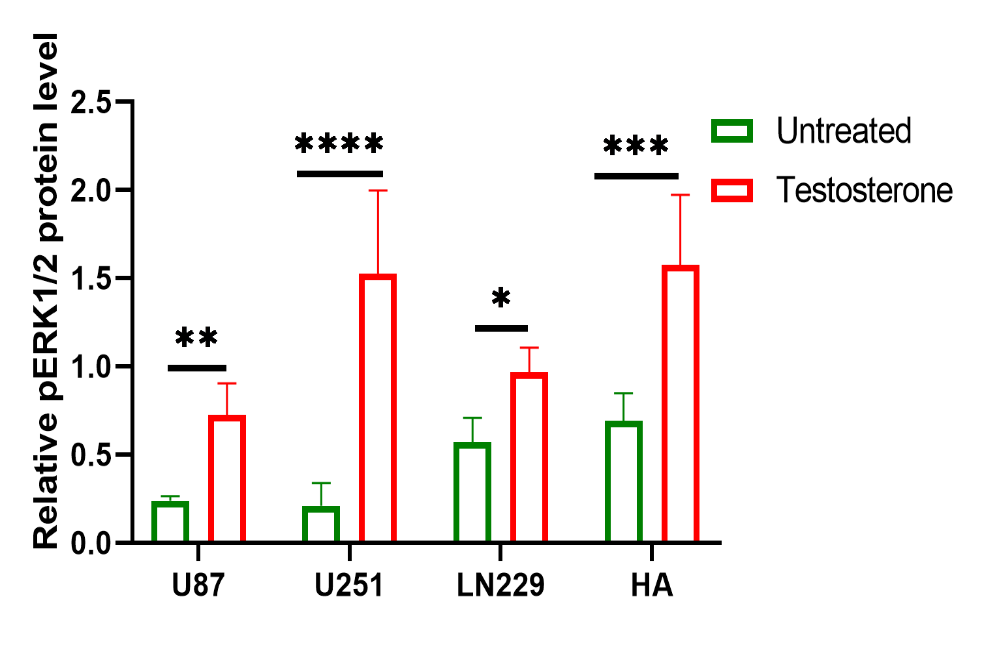


Western blot detection of ERK1/2 and pERK1/2 in U251, LN229, U87, and HA. The results showed that the ERK1/2 and pERK1/2 protein levels in the untreated glioma cell line was lower than in testosterone-treated glioma cell lines in which ERK1/2 and pERK1/2 protein levels significantly increased. the highest difference between the untreated and testosterone-treated pERK1/2 was observed in U251 and HA cells. *****p<0.05, ******p<0.01, *******p<0.001, and ********p<0.0001.

**Fig. S1K:**


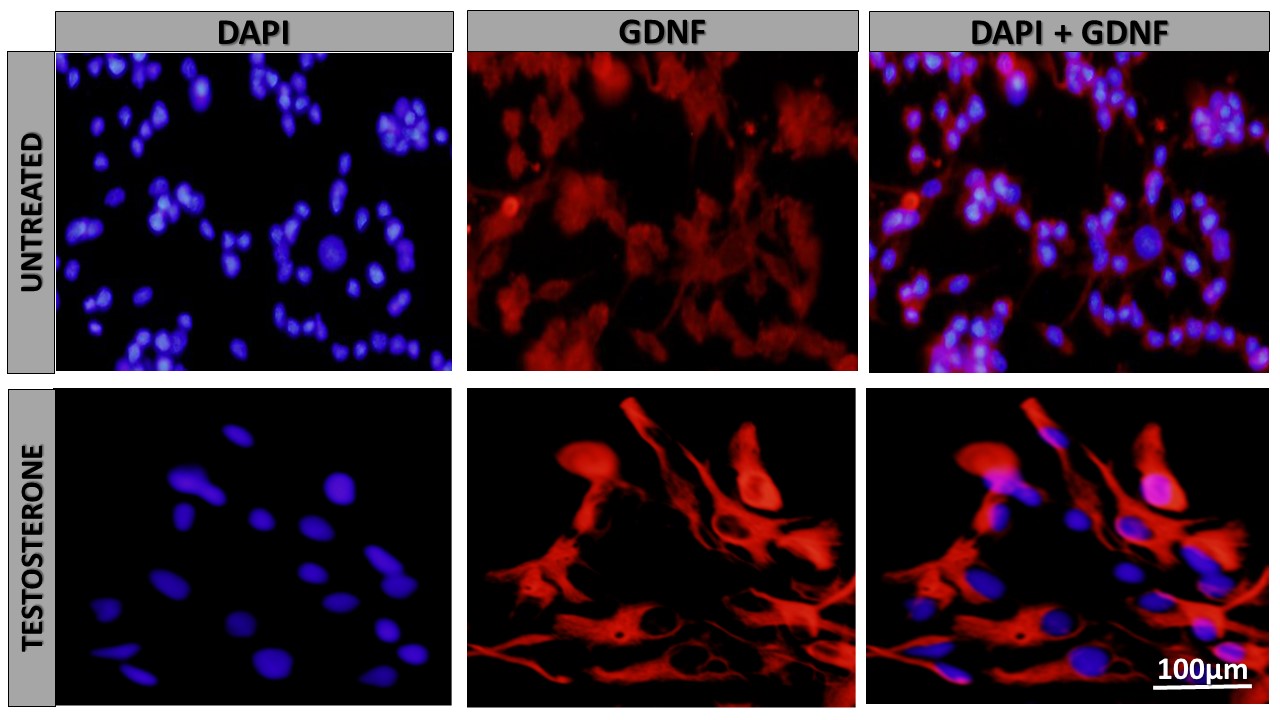


Immunofluorescence showing GDNF subcellular localization in human astrocyte (HA). The results showed that GDNF was found in the untreated group HA nucleus, while in testosterone treated group, GDNF is fount mainly in the cytoplasm.


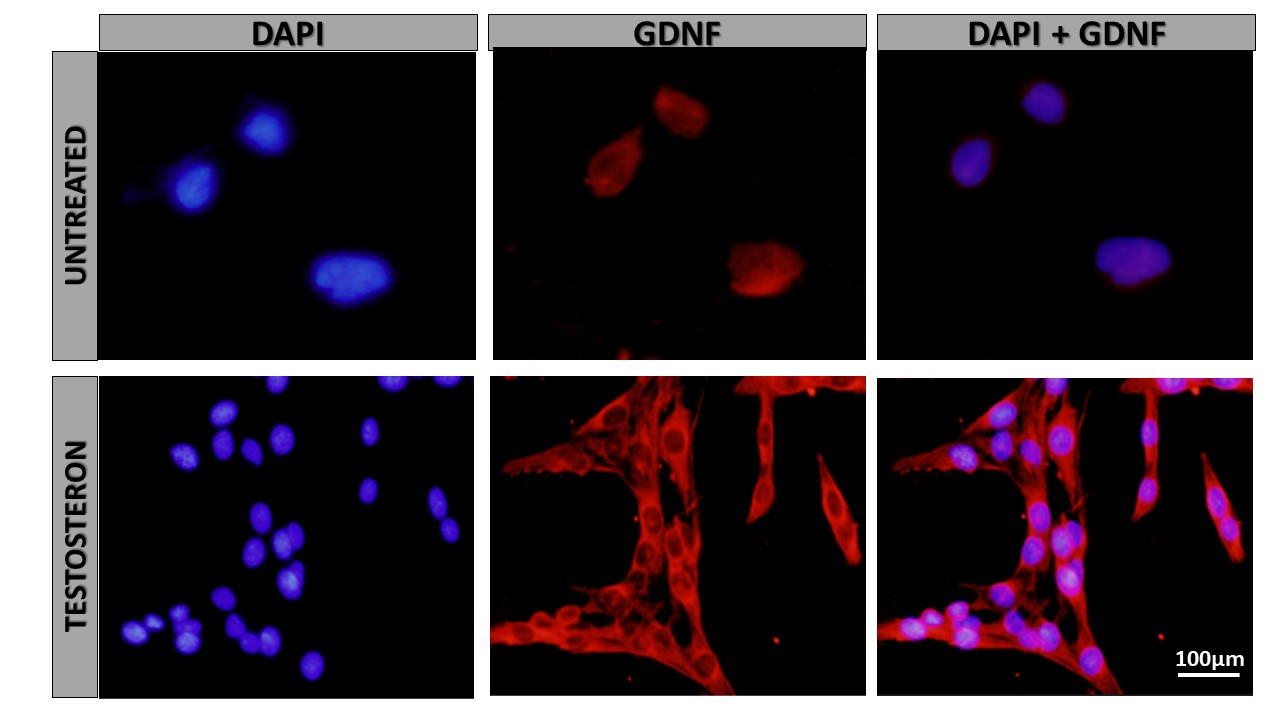
 Immunofluorescence showing GDNF subcellular localization in U251. The results showed that GDNF was found in the untreated group U251 nucleus, while in testosterone treated group, GDNF is fount mainly in the cytoplasm.


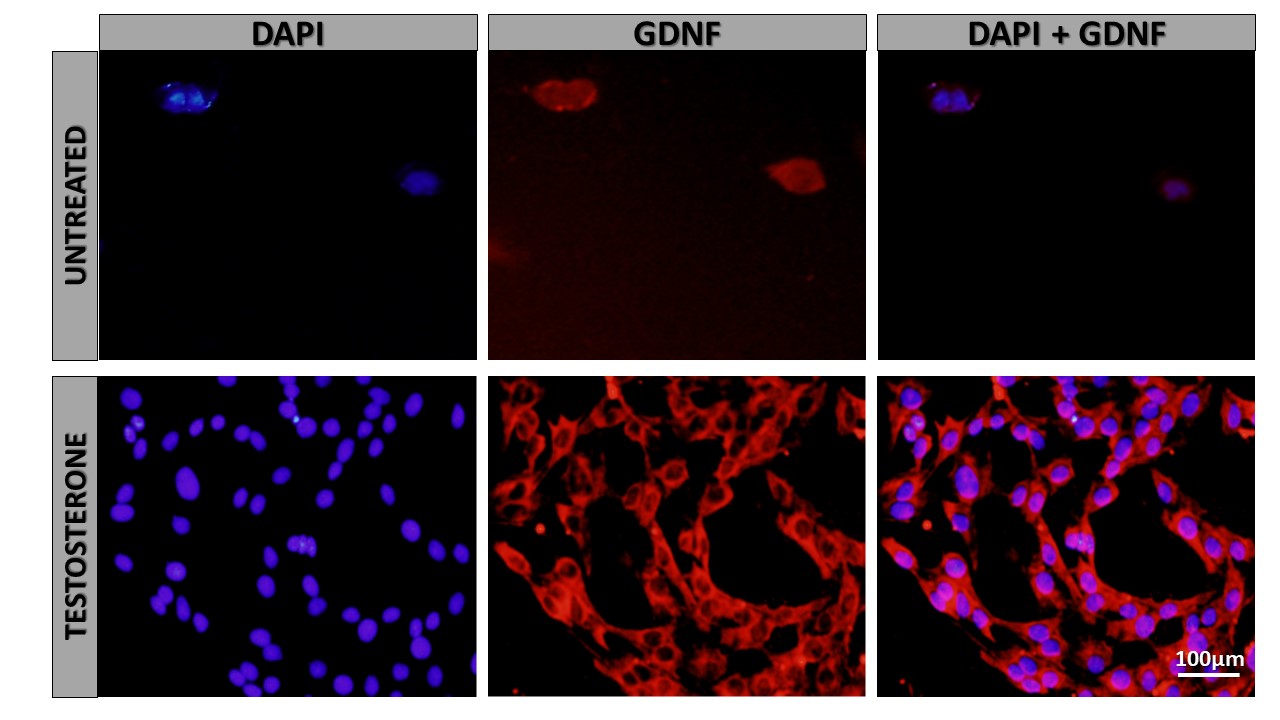


Immunofluorescence showing GDNF subcellular localization in LN229. The results showed that GDNF was found in the untreated group LN229 nucleus, while in testosterone treated group, GDNF is fount mainly in the cytoplasm.

**Fig. S1L**:


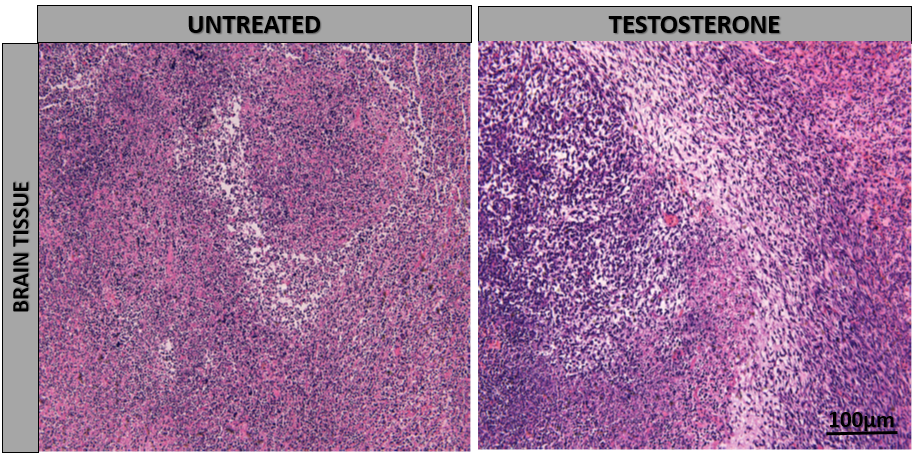


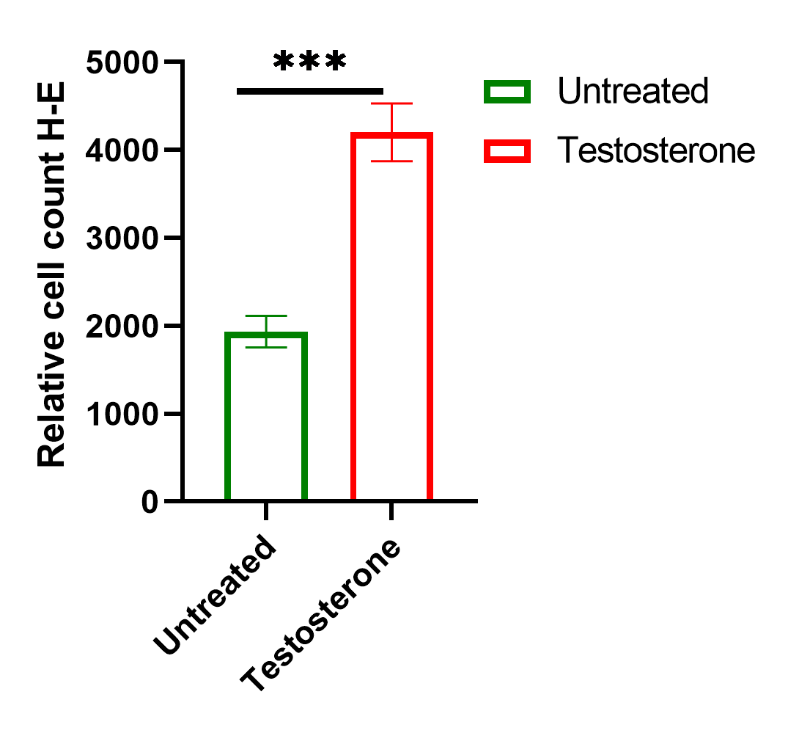


**Hematoxylin-eosin (H-E) of tumor tissue comparing inflammatory cell between the control (untreated) and testosterone treated groups.** The inflammatory cell is higher in testosterone treated tissue than the untreated tissue. *******P<0.001

**Fig. S1M**:


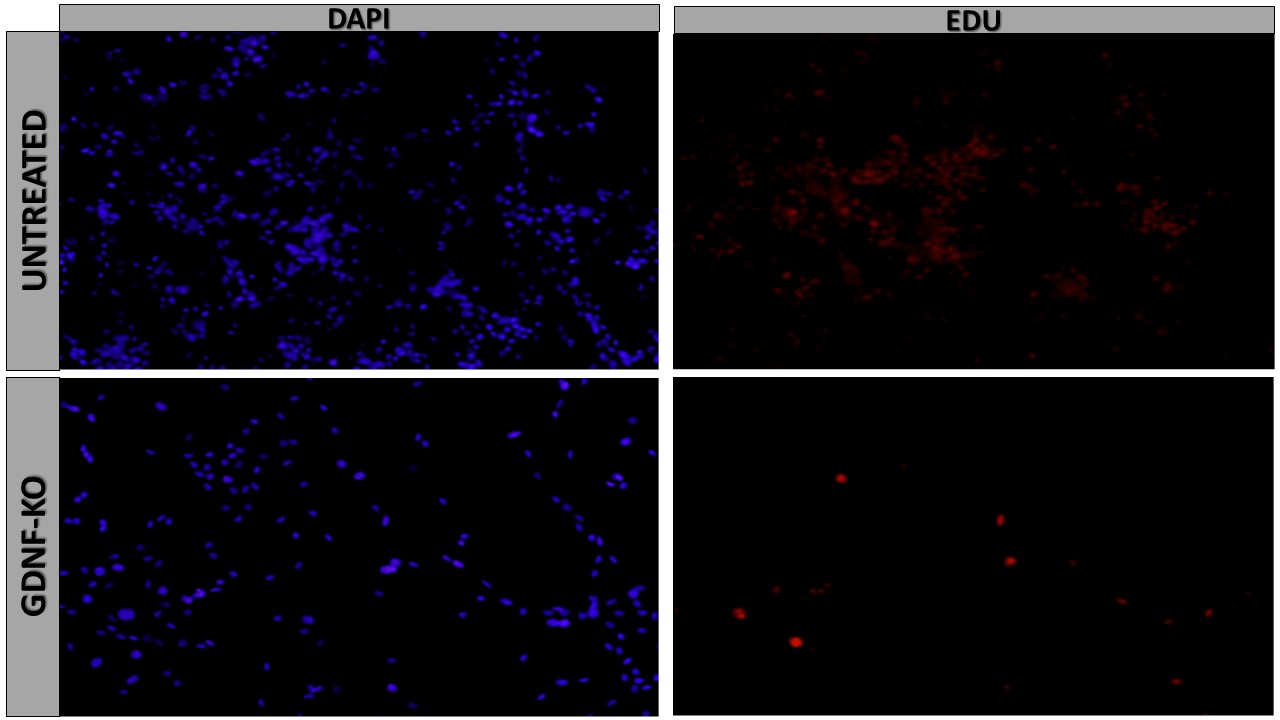


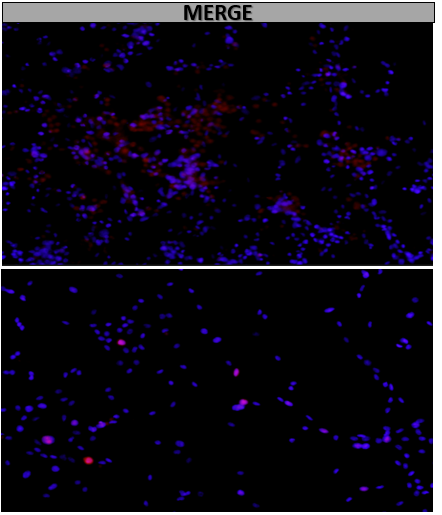

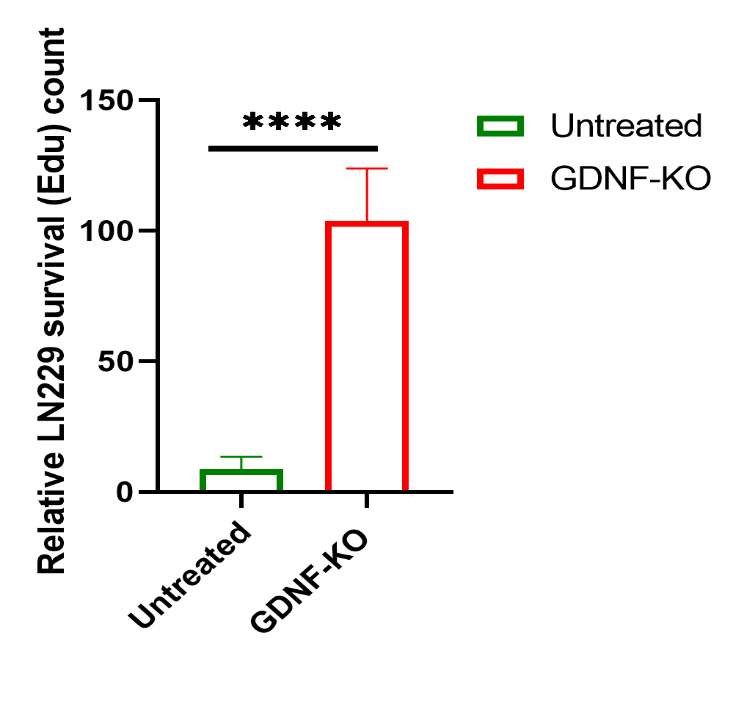


LN229 glioma cell line survival test via EDU assay. The staining of the LN229 glioma cell line with EDU solution showed that the Untreated LN229 glioma cell lines survival significantly increased compared with the GDNF-KO LN229 glioma cell lines, which showed a low survival. ********p<0.0001.

**Additional figures:**


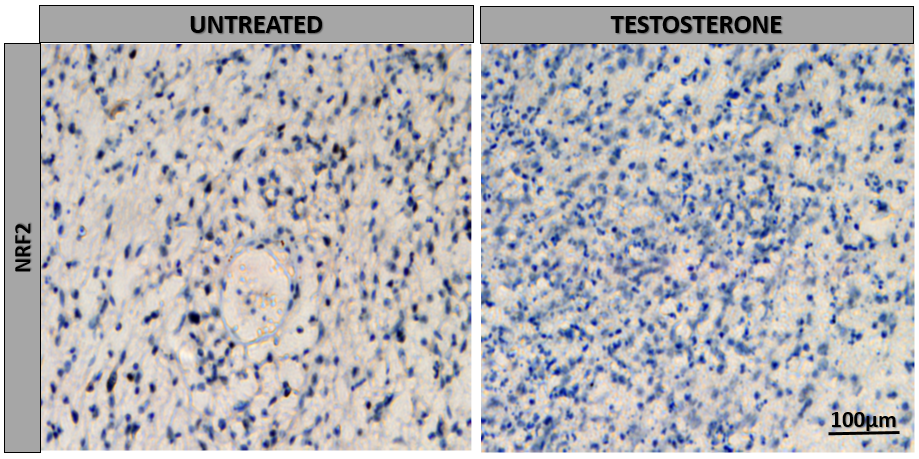


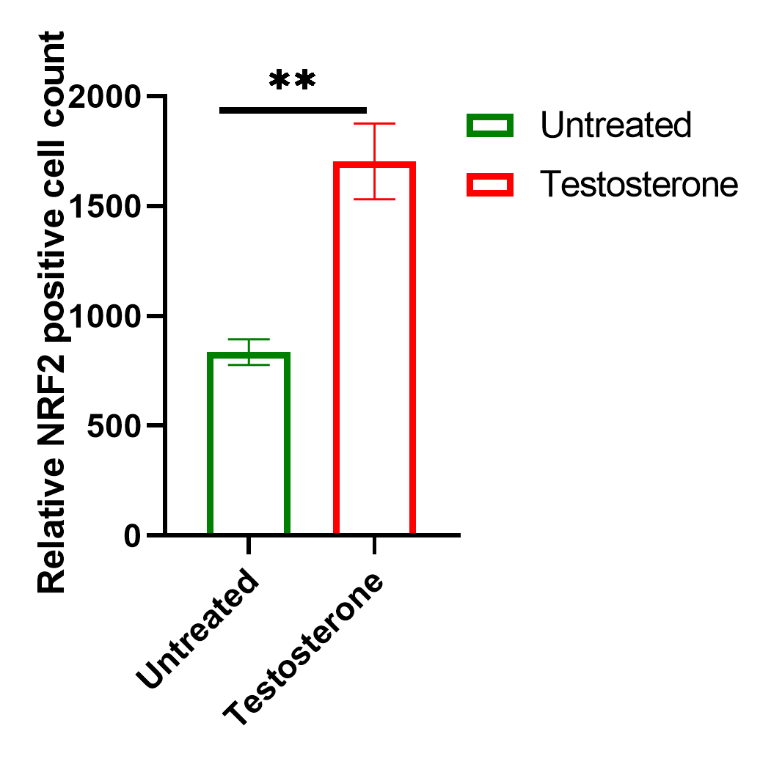


**Immunohistochemistry (IHC) of tumor tissue comparing NRF2 positive cells between the control (untreated) and testosterone-treated groups.** The NRF2 positive cell is higher in testosterone treated tissue than the untreated tissue. ******p<0.01


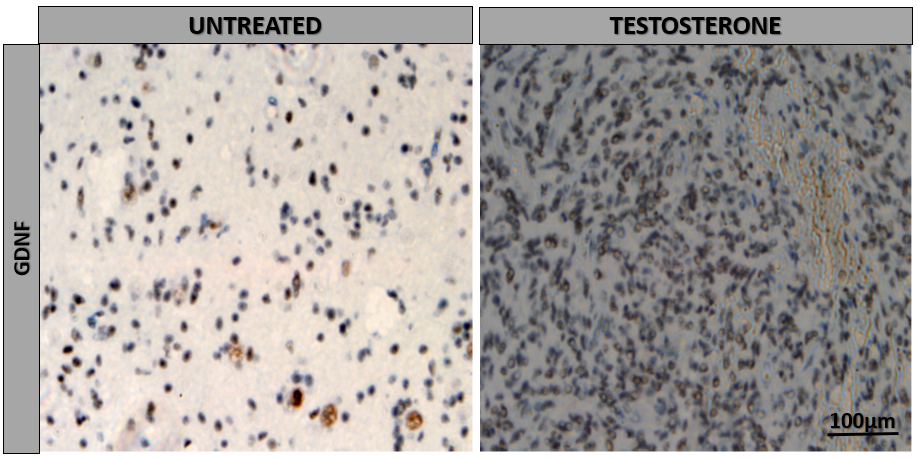


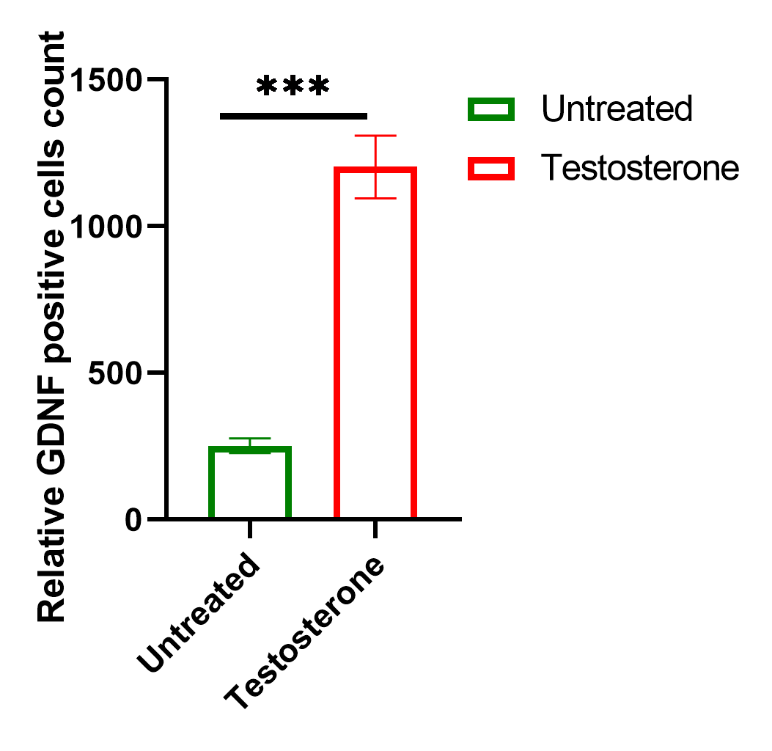


**Immunohistochemistry (IHC) of tumor tissue comparing GDNF positive cells between the control (untreated) and testosterone-treated groups.** The GDNF positive cell is higher in testosterone treated tissue than the untreated tissue. *******p<0.001


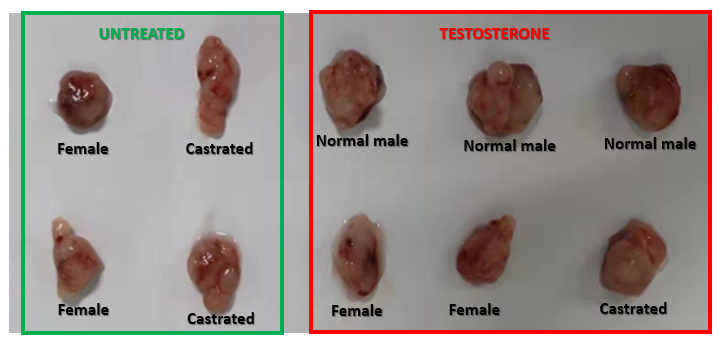


**Isolated tumor comparing tumor size between the control group (untreated) and testosterone treated normal male mice, castrated mice, and Female mice.** The biggest tumor was observed in normal male, followed by castrated male, and the female tumor was the smallest.
