# Supplementary figures and images for: Testosterone upregulates glial cell line-derived neurotrophic factor (GDNF) and promotes neuroinflammation to enhance glioma cell survival and proliferation
Source: Inflamm Regen. 2023 Oct 13;43:49. doi: 10.1186/s41232-023-00300-7 (PMC10571473; doi:10.1186/s41232-023-00300-7)

**Supplementary figure file**.

**Fig. S2**

**GDNF** :


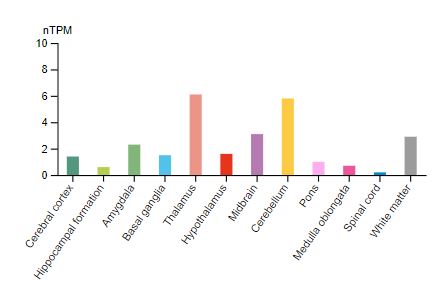


**SOX1** :


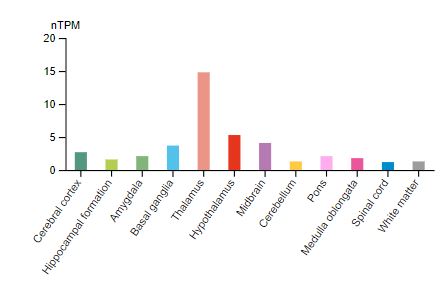


**Interleukine 6 (IL-6)** :


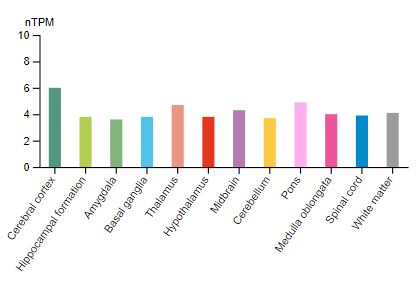


**NRF-2** :


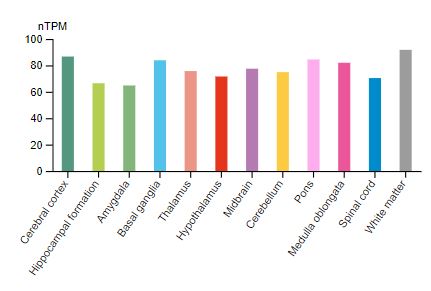


**CYCLOPHILIN A** :


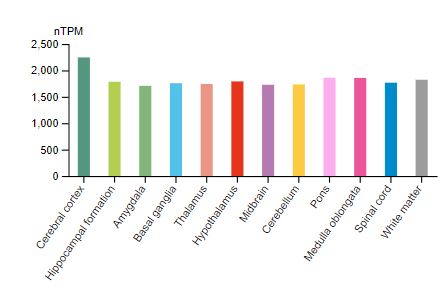


**COX2** :


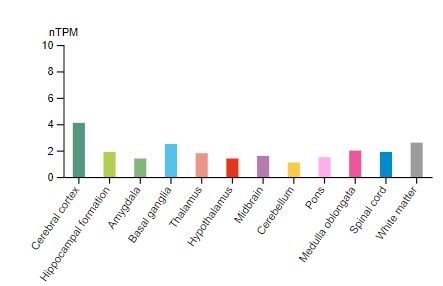


**ERK1/2** :


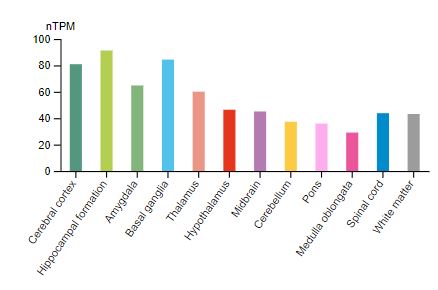

Supplement: Supplementary file 2 — Additional file 2: Figure S2. The protein atlas analysis indicated that GDNF, SOX1, cyclophilin-A, NRF2, ERK1/2, COX2, and IL-6 are highly detected in the thalamus and hypothalamus, whose activity relies on testosterone. [file 41232_2023_300_MOESM2_ESM.docx]
